# Simplified Head-to-Tail Cyclic Polypeptides as Biomaterial-Associated Antimicrobials with Endotoxin Neutralizing and Anti-Inflammatory Capabilities

**DOI:** 10.3390/ijms20235904

**Published:** 2019-11-25

**Authors:** Na Dong, Chensi Wang, Xinran Li, Yuming Guo, Xiaoli Li

**Affiliations:** 1 State Key Laboratory of Animal Nutrition, College of Animal Science and Technology, China Agricultural University, Beijing 100193, China; 2 The Laboratory of Molecular Nutrition and Immunity, Institute of Animal Nutrition, Northeast Agricultural University, Harbin 150030, China; ndong@neau.edu.cn (N.D.); s1650059155@gmail.com (C.W.); zl1571382260@gmail.com (X.L.); 3 Heilongjiang Key Laboratory of Molecular Design and Preparation of Flame Retarded Materials, College of Science, Northeast Forestry University, Harbin 150040, China; lixiaoli0903@nefu.edu.cn

**Keywords:** head-to-tail cyclic antimicrobial peptides, cell selectivity, membrane, bactericidal mechanism, skin inflammation

## Abstract

The therapeutic application of antimicrobial peptides (AMPs), a potential type of peptide-based biomaterial, is impeded by their poor antimicrobial activity and potential cytotoxicity as a lack of understanding of their structure–activity relationships. In order to comprehensively enhance the antibacterial and clinical application potency of AMPs, a rational approach was applied to design amphiphilic peptides, including head-to-tail cyclic, linear and D-proline antimicrobial peptides using the template (IR)_n_P(IR)_n_P (*n* = 1, 2 and 3). Results showed that these amphiphilic peptides demonstrated antimicrobial activity in a size-dependent manner and that cyclic peptide OIR3, which contained three repeating units (IR)3, had greater antimicrobial potency and cell selectivity than liner peptide IR3, DIR3 with D-Pro and gramicidin S (GS). Surface plasmon resonance and endotoxin neutralization assays indicated that OIR3 had significant endotoxin neutralization capabilities, which suggested that the effects of OIR3 were mediated by binding to lipopolysaccharides (LPS). Using fluorescence spectrometry and electron microscopy, we found that OIR3 strongly promoted membrane disruption and thereby induced cell lysis. In addition, an LPS-induced inflammation assay showed that OIR3 inhibited the pro-inflammatory factor TNF-α in RAW264.7 cells. OIR3 was able to reduce oxazolone-induced skin inflammation in allergic dermatitis mouse model via the inhibition of TNF-α, IL-1β and IL-6 mRNA expression. Collectively, the engineered head-to-tail cyclic peptide OIR3 was considerable potential candidate for use as a clinical therapeutic for the treatment of bacterial infections and skin inflammation.

## 1. Introduction

The continuous evolution of bacteria that has been accompanied by a reduced number of effective antibiotics has led to a global healthcare crisis [1]. The development of new antibiotics and novel approaches is critical for preventing an outbreak of drug-resistant bacteria, especially superbugs. Peptide-based biomaterials have been of increasing interest for use in the treatment antibionts, which has accelerated the development of nature-derived and synthetic peptides for therapeutic applications. Antimicrobial peptides (AMPs), endogenous immune components produced by various tissues of invertebrate and vertebrate animal, have been explored and used in a variety of biomedical applications, including as anti-infection agents, anticancer agents and biomedical coatings [2].

AMPs can selectively target and disintegrate bacterial membranes via electrostatic interactions and insertion into membrane lipid domains, leading to cell death; this is achieved through direct interaction with the cell membrane of bacteria, hence avoiding potential forms of resistance [3,4]. A recent study found that a new peptide derived from cathelicidin from *Bungarus fasciatus* was a competent candidate to be a novel antimicrobial compound for use against methicillin-resistant *Staphylococcus aureus* [5]. An in vivo study demonstrated that antiadhesive, antimicrobial peptide surface coatings can prevent bacterial adhesion and planktonic bacterial growth, thereby inhibiting catheter-associated infections in a murine urinary infection model [6]. However, there are technological hurdles impeding the therapeutic application of peptide-based biomaterials, including the high cost of isolation, potential systemic toxicity, instability and poor biocompatibility with host cells [7]; particularly, naturally secreted defenses could be compromised by natural peptides and their derivatives, possibly causing a serious public health problem. Therefore, the optimization of peptide molecular structures to enhance cell selectivity and anti-inflammatory ability and decrease the cost of production has become a principal challenge in the exploration of a new generation of antimicrobial drugs.

At present, more than 40 cyclic peptide drugs are applied in clinical practice with a great potential application effect [8]. AMPs with a restrained skeleton, especially a head-to-tail cyclic structure, can be utilized in developing novel antimicrobial drugs with increased activity [9]. A recent study found that rational design of head-to-tail cyclic peptides could be utilized to develop drug-like peptides as potent therapeutic Nrf2 activators [10]. Additionally, the cyclization of peptides can enhance their stability, resistance to exo- and (to some extent) endo-peptidases, binding affinity and selectivity towards target biomolecules; therefore, cyclic peptides have been actively investigated for use as biochemical tools and therapeutic agents [11]. In view of the condition-resistance stability of cyclic peptides and their high penetration efficiency, cyclic peptides are considered as ideal candidates for use as antibacterial drugs [12].

The most highly representative head-to-tail cyclic antimicrobial peptide is gramicidin S (GS) (cyclo(Val-Orn-Leu-DPhe-Pro)2), which is a cyclic decapeptide isolated from the bacterium *Bacillus brevis* [13]. GS has strong antimicrobial activity, especially towards Gram-positive bacteria and some pathogenic fungi. However, GS not only acts on bacterial membranes, but also on the membranes of mammalian cells such as erythrocytes [14]. For this reason it is limited in its use as an antibiotic in clinical medicine, the food industry and animal husbandry. The design strategies used for cyclic peptide therapeutics are generally limited by a poor understanding of sequence–structure relationships. Herein, we report the design of a simplified head-to-tail cyclic polypeptide as a biomaterial-associated antimicrobial, in order to tackle the problem of the high cytotoxicity of cyclic peptide-based drugs as well as to investigate the relationships between biological activity, conformation and modification. We designed a series of head-to-tail cyclic peptides, OIR1, OIR2 and OIR3, using the template sequence (IR)_n_P(IR)_n_P (*n* = 1, 2 and 3). The peptide sequences consist of the hydrophobic amino acid isoleucine (Ile; I) and the hydrophilic amino acid arginine (Arg; R). In addition, these cyclic peptides were decyclized to obtain linear counterpart peptides IR1, IR2 and IR3. In addition, in order to obtain antimicrobial peptides with high bacterial cell selectivity [15,16], we also substituted the L-Pro amino acids in IR1, IR2 and IR3 with D-Pro to generate the peptides DIR1, DIR2 and DIR3, respectively. The secondary conformations of the engineered peptides were characterized both in aqueous solution and in a simulated membrane environment using circular dichroism spectroscopy (CD). The antimicrobial activity of various salt ions and serum added at physiological concentration was measured using the minimum inhibitory concentration (MIC) method, and hemolytic activity and cytotoxicity was also determined. Peptide membrane interactions were investigated using fluorescence, flow cytometry and electron microscopy. We also developed a model of skin inflammation to explore the inhibitory effect of cyclic antimicrobial peptides on various inflammatory factors. This study had two main objectives: (1) to investigate the effect of peptides with varying lengths and secondary structures, including head-to-tail cyclic, decyclized and D-proline peptides, on antimicrobial potency and cell selectivity; and (2) to comprehensively evaluate the antibacterial potency and ability to inhibit skin inflammation of the engineered antimicrobial peptides, while developing synthetic peptide-based strategies to generate effective AMPs.

## 2. Results

### 2.1. Design and Characterization of the Peptides

In this experiment, cyclic, linear and D-proline antimicrobial peptides were designed based on the IR-rich template sequence (IR)_n_P(IR)_n_P (*n* = 1, 2 and 3). The β-sheet conformation created an amphiphilic structure between the hydrophobic Ile side chains existing in a nonpolar face of the cyclic peptide molecule and the cationic Arg side chains, which is considered to be essential for its bioactivity. By decyclizing the cyclic peptides, we obtained linear counterpart peptides IR1, IR2 and IR3. We replaced the L-Pro amino acids in IR1, IR2 and IR3 with D-Pro to obtain the peptides DIR1, DIR2 and DIR3, respectively. The secondary structures and molecular weights of the peptides were identified using mass spectrometry. The theoretical and the measured molecular weight, the net charge number, the retained time and the amino acid residue sequence of each engineered antimicrobial peptide are shown in Table 1. The peptide fidelity was first confirmed by MALDI-TOF MS as summarized in Figure S1. Results showed that there was a good agreement between the calculated and measured molecular weights, indicating that all of the antimicrobial peptides were synthesized accurately. The hydrophobicity of the peptides in solution is dependably mirrored by the HPLC retention times [17]. In this experiment, the retention times of OIR1, OIR2, OIR3, IR1, IR2, IR3, DIR1, DIR2 and DIR3 were 10.32, 10.66, 12.64, 10.28, 10.58, 12.4, 10.44, 10.60 and 12.82 min, respectively, indicating that the hydrophobicity of OIR3, IR3 and DIR3 were higher than those of shorter peptides.

### 2.2. Circular Dichroism Spectroscopic Analysis

**Figure 1:**
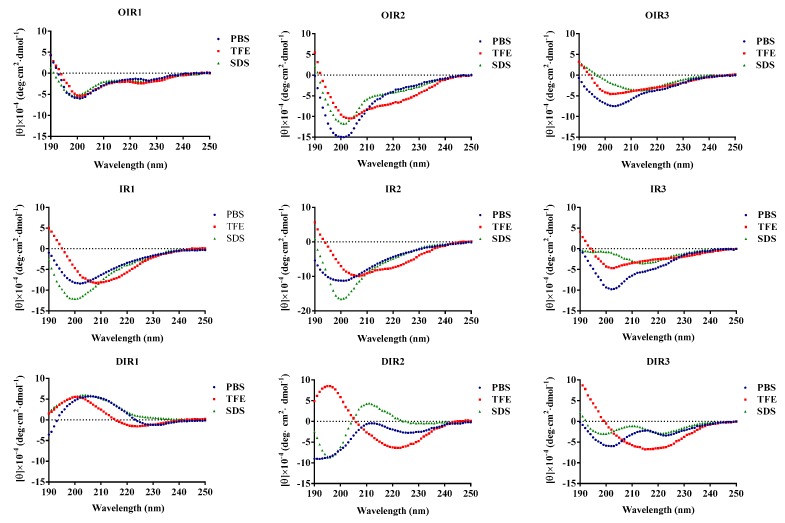
The circular dichroism (CD) spectra of the engineered peptides. The peptides were dissolved in 10 mM sodium phosphate buffer (pH 7.4), 30 mM SDS and 50% (*v/v*) trifluoroethanol. The mean residue ellipticity was plotted against wavelength. The values from three scans were averaged per sample, and the peptide concentrations were fixed at 150 μM.

CD spectroscopy of the all engineered peptides revealed their secondary structures in aqueous solution and in a membrane-mimetic environment. The CD spectrum of the engineered peptides were determined in different liquid environment including 10 mM sodium phosphate buffer (pH 7.4), 50% TFE or 30 mM of SDS micelles (Figure 1). In aqueous solution, each of the engineered peptides, with the exception of DIR1, appeared to assume an unordered conformation. In the presence of 50% TFE, DIR1 and DIR2 more tended to be in the β-sheet conformation. The CD spectrum with the maximum value of near 190 nm and the minimum value near 216 nm indicated that DIR3 showed more β-hairpin structure [18]. Other peptides (except DIR1, DIR2 and DIR3) showed evidence of the presence of a sub-population displaying a β-hairpin conformation that was distinguished by a negative ellipticity near 205 nm and a crossover at 200 nm [19]. In the presence of 30 mM anionic SDS, IR1, IR2 and IR3 each displayed characteristics of an unordered conformation, while OIR1, OIR2, OIR3 and DIR3 each appeared to have a population that assumed a β-hairpin conformation as described above [19]. DIR1 assumed an obvious β-sheet conformation and the DIR2 spectra indicated the presence of a significantly greater number of β-turns.

### 2.3. Antimicrobial and Hemolytic Activities and Selectivities

**Figure 2:**
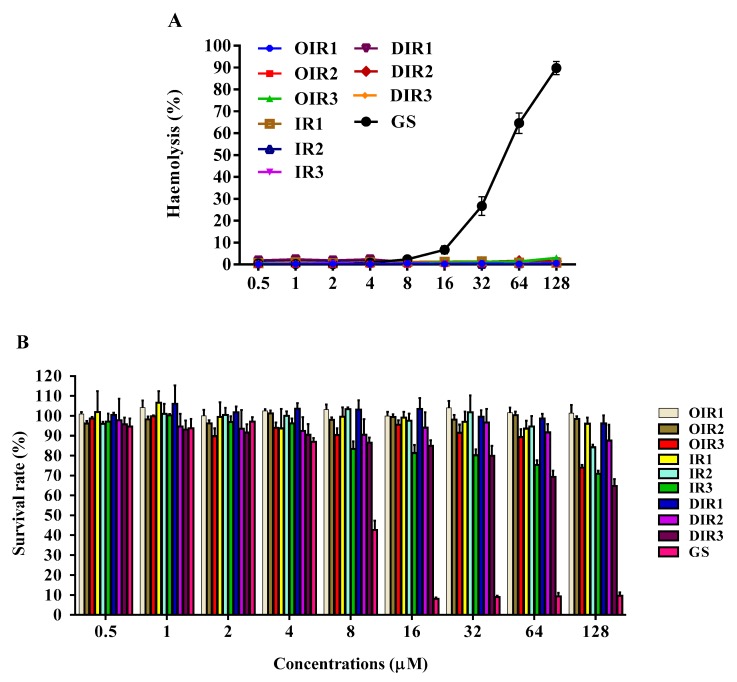
(**A**) Hemolytic activity of the engineered peptides against human red blood cells (hRBCs). The hRBCs were incubated with equal amounts of the respective peptides dissolved in PBS for 1 h at 37 °C. (**B**) Cytotoxicity of the engineered peptides against RAW264.7. The cells were incubated with various concentrations (0.5–128 μM) of peptides for 24 h, and the cell viability was determined by the MTT assay. Data shown are three independent experiments.

As shown in Table 2, the antibacterial activity of the engineered peptides was detected against both Gram-positive bacteria and Gram-negative bacteria. Results showed that the MIC of OIR1, IR1 and DIR1 in the presence of Gram-positive and Gram-negative bacteria was greater than 128 μM. As the length of the peptide chain increased, its antimicrobial activity also increased. Peptides containing three repeating units (IR3) had the highest antimicrobial activity, especially the cyclic peptide OIR3. The antimicrobial activity of IR3 and DIR3 was lower than that of OIR3, indicating that the linearization of the cyclic peptide OIR3 and the substitution with D-proline decreased the peptides antimicrobial activity. Cyclic peptide OIR3 displayed the greatest antibacterial activity, with at GM MIC of 3.5–4.0 µM, comparable to that of the broad-spectrum antibiotics GS (GM = 2–6.1 µM), chloramphenicol (CM; GM = 18.4–22.6 µM), colistin sulfate (CS; GM = 0.8–76.1 µM) and penicillin sodium (PS; GM = 13.5–24.3 µM). The results of hemolytic activities of the peptides against human erythrocytes (Table 2 and Figure 2A) confirm that none of the engineered peptides displayed hemolytic activity, even at the highest concentration of 128 μM, while the control cyclic peptide GS exhibited a high level of hemolytic activity.

To investigate the cell selectivity of novel antimicrobials, the total selectivity index (TSI) was calculated as the ratio of the minimal hemolysis concentration (MHC) to the geometric mean (GM) of the MIC values (Table 2). Cyclic peptide OIR3 displayed the greatest TI (69.2), which was four-fold greater than that of the linear counterpart peptide IR3 (11.3) or D-Pro peptide DIR3 (17.3). This demonstrated that the cyclic peptide OIR3 had a greater selectivity towards microorganisms versus erythrocytes, suggesting that it may be used during a larger treatment window.

### 2.4. Cytotoxicity

The results of cytotoxicity testing of the engineered peptides displayed that the length of the peptide slightly affected its cytotoxic activity (Figure 2B). The survival rates of RAW264.7 cells exposed to OIR1, OIR2 and OIR3 were 99%, 95% and 70%, respectively, at the highest concentration of 128 μM; with a decrease in the OIR3 concentration, the cytotoxicity decreased, indicating that OIR3 showed almost no toxicity towards mammalian cells. The survival rates of cells exposed to the linear peptide IR3 at the maximum concentration was 70%, indicating that linearization of the cyclic peptide OIR3 had no effect on its toxicity in cells, while the cell survival upon exposure to DIR3 at the maximum concentration was 65%, indicating that the presence of D-proline slightly increased the toxicity of the peptide. In line with its MHC value, the control peptide GS was highly toxic towards mammalian cells.

### 2.5. Salt Sensitivity and Serum Stability

Based on the MIC and MHC values, the salt sensitivity of peptides was detected by adding salt ions of varying physiological concentrations. The addition of monovalent (Na^+^, NH_4_^+^ and K^+^), divalent (Mg^2+^, Ca^2+^, and Zn^2+^) and trivalent cations (Fe^3+^) had little effect (Table 3). The antibacterial activity of OIR3 against *Escherichia coli (E. coli)* and *S. aureus* was slightly reduced in the presence of Na^+^ and Ca^2+^. Interestingly, the antibacterial activity of IR3 against *S. aureus* was 1–2 times stronger in terms of its MIC value, indicating that the antibacterial activity of the IR3 by the linearization of OIR3 is more susceptible to salt ions.

To further evaluate the serum stability of the engineered peptides, their antimicrobial activity against Gram-negative *E. coli* and Gram-positive *S. aureus* in the presence of 25% or 50% serum were measured (Table 3). The results displayed that the MICs of the engineered peptides were increased to varying degrees. The serum had a slight effect on the antimicrobial activity of OIR3 and DIR3, while IR3 and GS lost most of their antibacterial activity against *E. coli* and *S. aureus.*

### 2.6. Endotoxin Neutralization Activities

**Figure 3:**
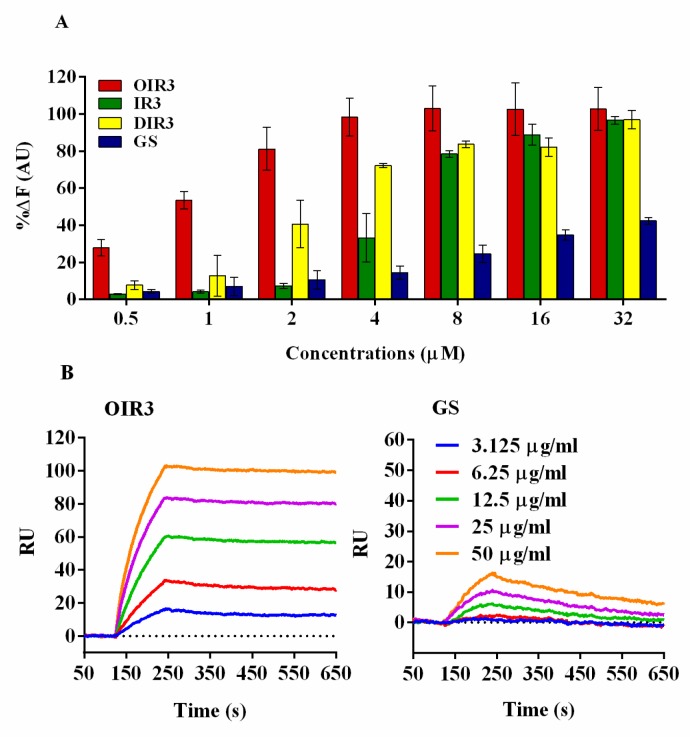
(**A**) The combining ability of OIR3 and gramicidin S (GS; 0.5 μM, 1 μM, 2 μM, 4 mM, 8 μM, 16 μM and 32 μM) with lipopolysaccharide (LPS). (**B**) SPR spectroscopy of the interaction kinetics of the peptides and LPS. The peptides were immobilized on a CM5 sensor chip as a ligand, and LPS was diluted in a series of concentrations (3.125, 6.25, 12.5, 25 and 50 μg/mL). Data shown are three independent experiments.

Lipopolysaccharide (LPS), a main component of the endotoxin derived from the outer membrane of Gram-negative bacteria, plays a role in systemic inflammatory response syndrome. Previous studies demonstrated that AMPs could bind LPS to gain access to bacterial membranes and facilitate subsequent membrane disruption or translocation into bacteria [22,23]. We determined the LPS-binding ability of the engineered peptides OIR3, IR3, DIR3 and GS with a fluorescence-based displacement assay with BODIPY-TRcadaverine and polymyxin B, which has well-known strong LPS-binding capability, as a reference compound. As shown in Figure 3A, the engineered peptides OIR3, IR3, DIR3 and GS displayed a dosage-dependent increase in fluorescence intensity, clearly demonstrating their ability to bind to LPS. Of all of the engineered peptides, the binding ability of the cyclic peptide OIR3 had the strongest binding ability for IR3 and DIR3. OIR3 had 81% LPS-binding activity at low peptide concentration (2 μM) and almost completely neutralized the LPS at 4 μM (98%). At all concentrations tested, the combining ability of GS with LPS was relatively low, and the combining ability even at the highest concentration of 32 μM was lower than that of OIR3 at 1 mM.

We used Surface Plasmon Resonance (SPR) spectroscopy to further demonstrate the ability of OIR3 and GS to bind to LPS. As shown in Figure 3B, LPS rapidly binds to immobilized OIR3 and had a significant metrological dependency on the LPS concentration (3.125–50 µg/mL), while the control peptide GS demonstrated weak binding to LPS at high concentrations and almost no binding at the low concentrations (3.125 and 6.25 µg/mL).

### 2.7. Outer Membrane Permeability Assay

**Figure 4:**
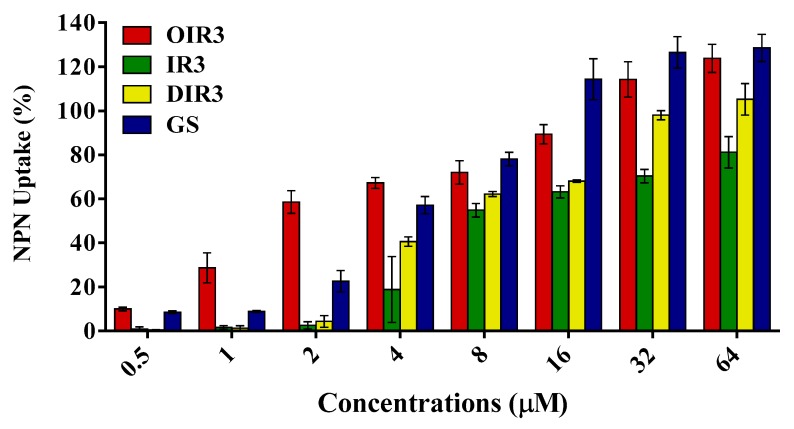
Outer membrane permeabilization assays of the engineered peptides. A 2 mL volume of *E. coli* UB1005 cells, diluted to 10^5^ CFU/mL in 5 mM sodium HEPES buffer, was added to a quartz cuvette containing NPN to give a final concentration of 10 μM. Aliquots of peptides were added to the cuvette, and the fluorescence was recorded until there was no further increase in fluorescence. Data shown are three independent experiments.

The above results displayed that the cyclic peptide OIR3 had higher antibacterial activity and stability than other engineered peptides, and its antibacterial mechanism was further examined by evaluating its interaction with the cell membrane of bacterial. The hydrophobic fluorescent probe NPN is usually blocked by the outer membranes of bacterial cells and quenched under aqueous conditions, but it is taken up if the outer membranes of bacterial cells are permeabilized and shows intense fluorescence intensity in a hydrophobic environment. Results showed that the engineered peptides could permeabilize the outer membrane of Gram-negative bacteria and a concentration-dependent manner. The outer membrane permeability of OIR3 was more than 70% at concentrations greater than 4 μM, and higher than that of the control peptide GS (Figure 4).

### 2.8. Inner Membrane Permeability Assay

**Figure 5:**
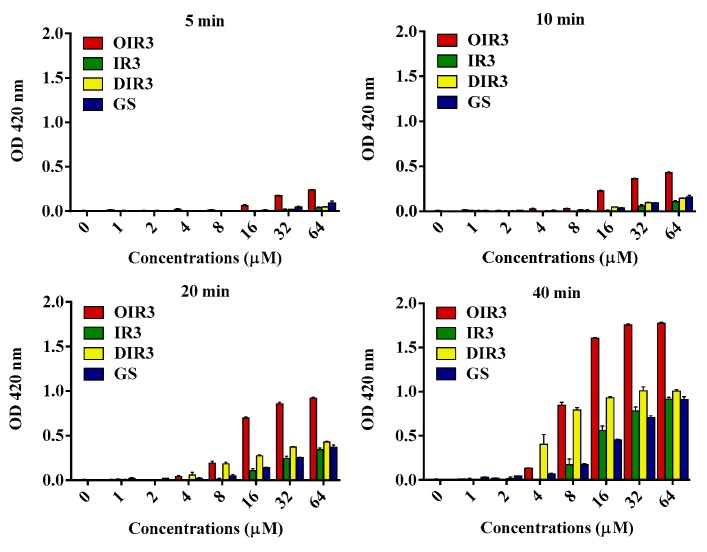
Inner membrane permeability of the engineered peptides. Hydrolysis of ONPG due to the release of cytoplasmic β-galactosidase of *E. coli* UB1005 treated with peptides OIR3, IR3, DIR3 and GS at a series of different time (5 min, 10 min, 20 min and 40 min) and different concentration (1μM, 2 μM, 4 μM, 8 μM, 16 μM, 32 μM and 64 μM) and measured spectroscopically at an absorbance of 420 nm as a function of time.

An o-nitrophenol-β-D-galactoside (ONPG) assay was performed to assess the inner membrane permeability of the engineered peptides (Figure 5). The results revealed that engineered peptide OIR3, IR3, DIR3 and GS showed inner membrane permeability in a time- and dosage-dependent manner. The cyclic peptide OIR3 had a higher endomembrane permeability activity than IR3, DIR3 and GS.

### 2.9. Cytoplasmic Membrane Electrical Potential Measurement

**Figure 6:**
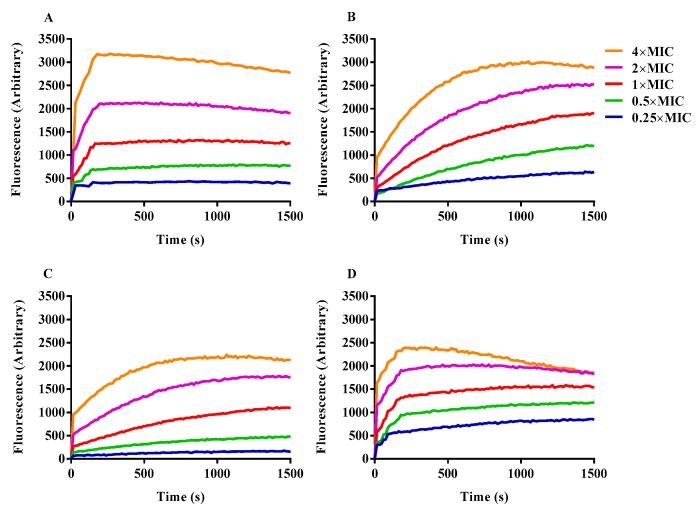
Cytoplasmic membrane depolarization of *E. coli* UB1005 was measured by using the membrane potential-sensitive dye, DiSC*3*-5 treated with the engineered peptides OIR3 (**A**), IR3 (**B**), DIR3 (**C**) and GS (**D**) at a series of different concentration 0.25× MIC, 0.5× MIC, 1× MIC, 2× MIC and 4× MIC.

A membrane potential-dependent probe (3, 3′-dipropylthiadicarbocyanine iodide; DiSC3-5) was adopted to evaluate the ability of engineered peptides to depolarize the bacterial cytoplasmic membrane. When the cytoplasmic membrane is permeable and destroyed, the membrane potential is eliminated, and released DiSC3-5 caused an increase in fluorescence. All of the detected peptides depolarized the cytoplasmic membrane of bacterial cells in a dosage-dependent manner (Figure 6). Compared with the other antimicrobial peptides, OIR3 induced cell membrane depolarization faster and more strongly.

### 2.10. Flow Cytometry Analysis

**Figure 7:**
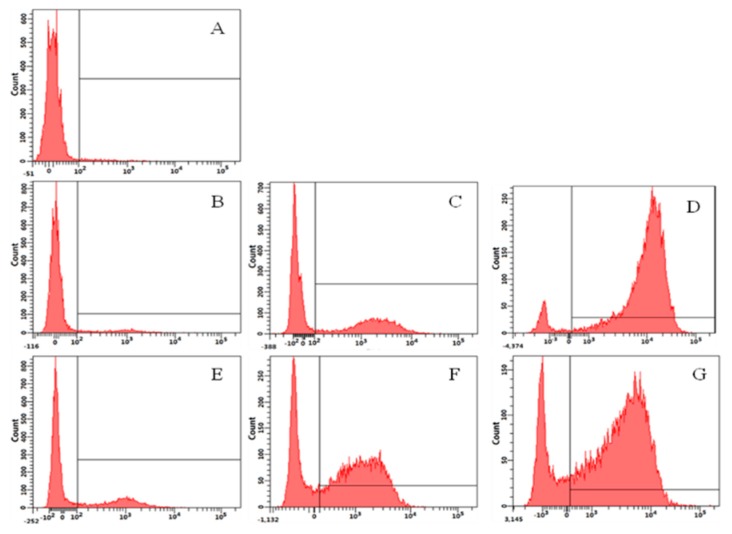
Flow cytometric analysis. Exponential phase *E. coli* cells were treated with GS, OIR3 and cellular fluorescence was analyzed by flow cytometry. The increments of the log fluorescence signal represent PI uptake resulting from peptide treatment. (**A**) No peptide, negative control; (**B**) GS (1/2× MIC, 2 µM); (**C**) GS (1× MIC, 4 µM); (**D**) GS (2× MIC, 8 µM); (**E**) OIR3 (1/2× MIC, 1 µM); (**F**) OIR3 (1× MIC, 2 µM) and (**G**) OIR3 (2× MIC, 4 µM).

The fluorescent dye Propidium Iodide (PI) can enter a dead cell and combine with DNA to produce red fluorescence; subsequently, flow cytometry can be used to detect the intensity of the fluorescence release and determine the proportion of dead cells resulting from peptide exposure. Analysis of the results displayed that the control sample (A) contained only 2.2% PI-positive cells. The percentage of PI-positive cells due to exposure to OIR3 was 25% (E, 1/2× MIC), 62% (F, 1× MIC) and 76.4% (G, 2× MIC). Treatment with GS resulted in 7.3% (B, 1/2× MIC), 37.8% (C, 1× MIC) and 93.3% (D, 2× MIC) PI-positive cells (Figure 7). Our data found that OIR3 caused a bigger accumulation of PI than GS at ≤1× MIC, while GS caused greater accumulation than OIR3 at 2× MIC.

### 2.11. Scanning Electron Microscopy (SEM) and Transmission Electron Microscopy (TEM)

**Figure 8:**
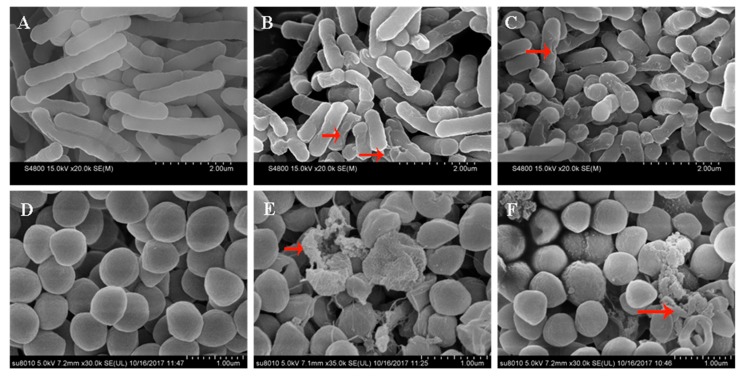
Scanning electron micrographs of *E. coli* 25,922 and *S. aureus* ATCC 29,213 treated with GS and OIR3. SEM micrographs of *E. coli*: (**A**) control, no peptides; (**B**) OIR3-treated, 2 μM (**C**) GS-treated, 4 μM. SEM micrographs of *S. aureus*: (**D**) control, no peptides; (**E**) OIR3-treated, 4 μM and (**F**) GS-treated, 2 μM. Scanning electron micrographs *E. coli* and *S.* *aureus* bacteria in mid-logarithmic growth were treated with peptides at 1× MIC for 1 h. The red arrow indicates the difference from the control group.

**Figure 9:**
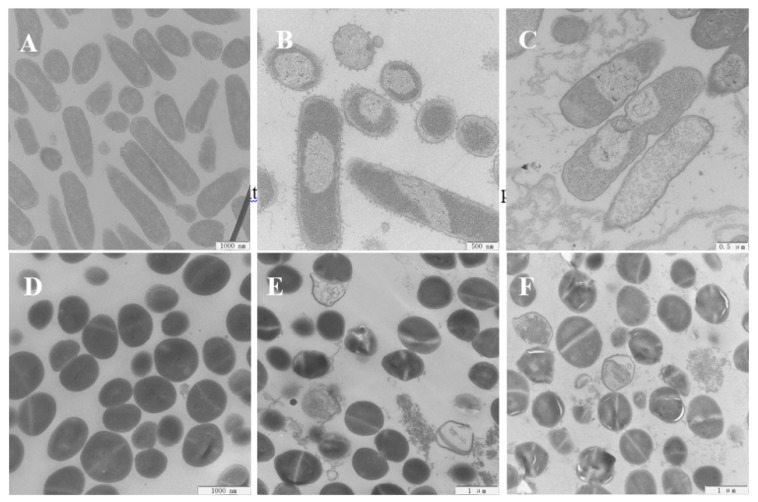
Transmission electron microscopy of *E. coli* 25,922 and *S. aureus* ATCC 29,213 treated with GS and OIR3. TEM micrographs of E. coli: (**A**) Control, no peptides. The scale is 1000 nm. (**B**) OIR3-treated, 2 μM. The scale is 500 nm. (**C**) GS-treated, 4 μM. The scale is 500 nm. TEM micrographs of S. *aureus* at the scale of 1000 nm: (**D**) Control, no peptides. (**E**) OIR3-treated, 4 μM and (**F**) GS-treated, 2 μM. TEM micrographs *E. coli* and *S. aureus* bacteria in mid-logarithmic growth were treated with peptides at 1× MIC for 1 h.

The antimicrobial mechanisms utilized by the engineered peptides against *E. coli* 25,922 and *S. aureus* 29,213 cells were further analyzed by using SEM to study the transformation of morphological characteristics. The control cells, which were not treated with any peptides, displayed brilliant and smooth membrane surfaces (Figure 8A,D). However, when the bacterial cells are exposed to OIR3 and GS at 1× MIC, the *S. aureus* and *E. coli* cell membranes became rough and wrinkled and appeared destroyed. In order to further observe the changes in morphologic and internal microstructure of bacteria cell, TEM was adopted and the results that the control *E. coli* and *S. aureus* cells were smooth and complete (Figure 9A,D). After 1 h of treatment with both OIR3 and GS, the morphology of bacteria had undergone significant changes and there was evidence of cell membrane rupture and cytoplasmic leakage; in particular, the bacterial surfaces of cells treated with OIR3 appeared to have formed vesicular protuberances.

### 2.12. Swimming Motility Assays

**Figure 10:**
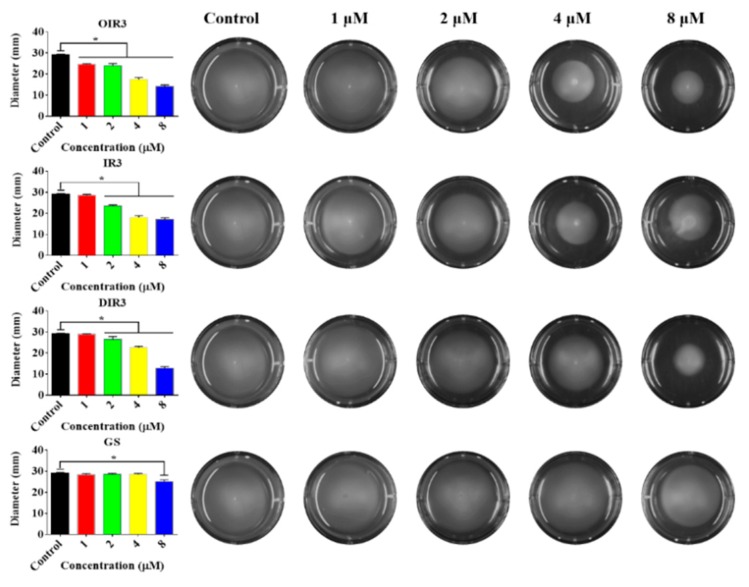
Swimming motility of *E. coli* ATCC 25,922 treated with OIR3, IR3, DIR3 and GS. Swim plates were prepared using 0.3% agar and inoculated from overnight cultures standardized to an optical density at 600 nm of 1.0. Images were taken after 20 h incubation at 37 °C. Data shown are the mean ± SD of three independent experiments. Bars with “*” represent significantly different mean values with *p* < 0.05.

The flagella exist on the exterior of the cell wall of Gram-negative bacteria, which can impel the bacterial motility and chemotaxis that are crucial to the process of infection for many pathogenic bacteria. As shown in Figure 10, the engineered peptides OIR3, IR3 and DIR3 can significantly inhibit the swimming motility of *E. coil* cells at concentrations of 4 μM and 8 μM. The control peptide GS produced almost no inhibition of motility at all tested concentrations.

### 2.13. Anti-Inflammatory Activities

**Figure 11:**
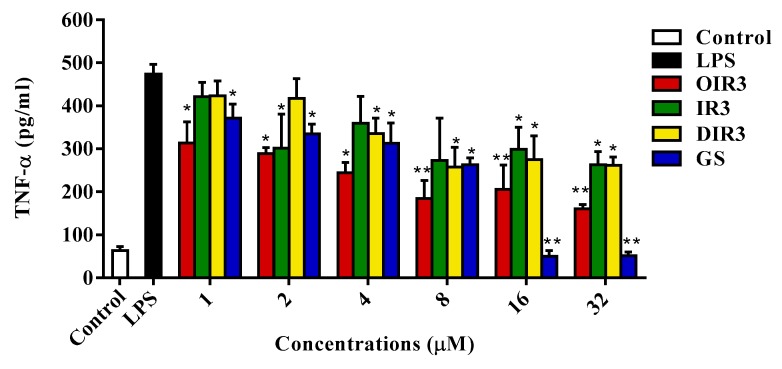
The inhibitory effects of OIR3, IR3, DIR3 and GS on LPS-stimulated TNF-α production in RAW264.7 cells. The levels of TNF-α in the cytoplasm were determined by ELISA using a commercial kit for mouse TNF-α. Data shown are the mean ± SD of three independent experiments. “*” represent significantly different mean values with *p* < 0.05. “**” represent significantly different mean values with *p* < 0.01.

LPS-stimulated TNF-α production in the cytoplasm of RAW264.7 cells was determined using ELISA of mouse TNF-α performed with a commercial kit (Boster, Wuhan, China; Figure 11). The cyclic peptide OIR3 more strongly inhibited the expression of TNF-α in a dosage-dependent method than did either the linear peptide IR3 or the D-Pro-containing peptide DIR3 at all tested concentrations (*p* < 0.01). At low concentrations (peptide concentration below 8 μM), the cyclic peptide OIR3 had a stronger ability to inhibit the production of TNF-α than the control cyclic peptide GS. At high concentrations (peptide concentrations above 8 μM), the control loop peptide GS had cytotoxicity resulting in cell death and resulting in lower expression of TNF-α.

### 2.14. Effects of Peptides on Oxazolone-Induced Skin Inflammation in Mice

**Figure 12:**
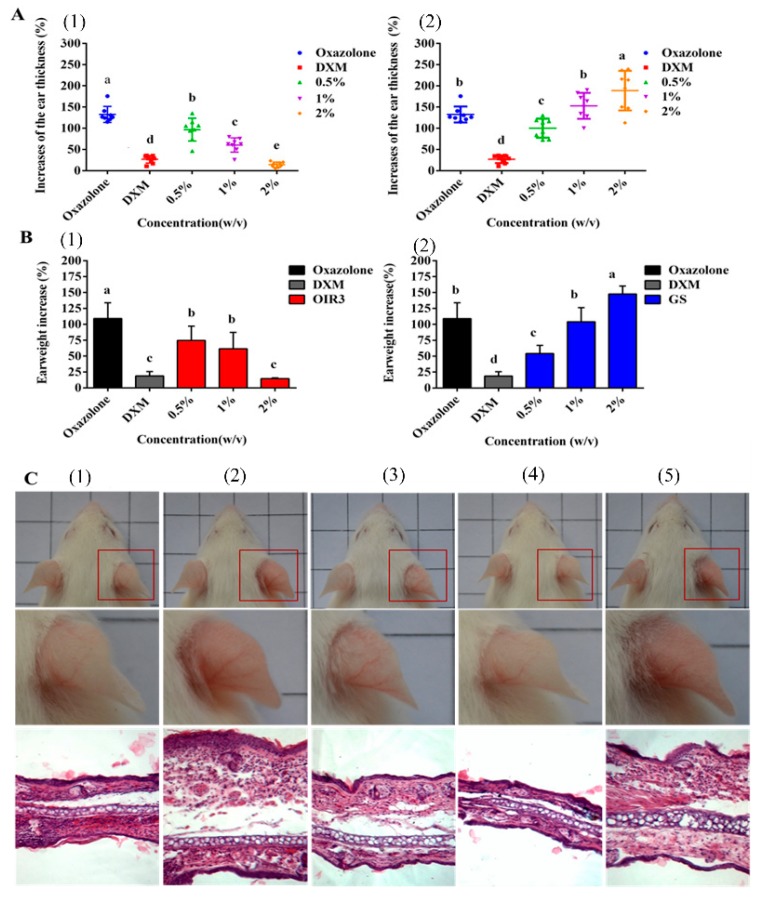
(**A**) Treatment with OIR3 attenuates oxazolone-induced ear skin inflammation of mice. Changes of ear thickness (1, OIR3; 2, GS) and (**B**) weights of ear punches (1, OIR3; 2, GS) were evaluated. (**C**) Gross findings of mice (upper panels) and microscopic findings of ear skin after H&E staining (lower panels) at 24 h after treatment. (1), negative control (95% ethanol); (2), positive control (oxazolone); (3), 1% DXM (*w/v*); (4), 2% OIR3 (*w/v*) and (5), 2% GS (*w/v*). The error bars represent mean ± SD. Data shown are the mean ± SD of three independent experiments. Bars with different letters represent significantly different mean values with *p* < 0.05.

**Figure 13:**
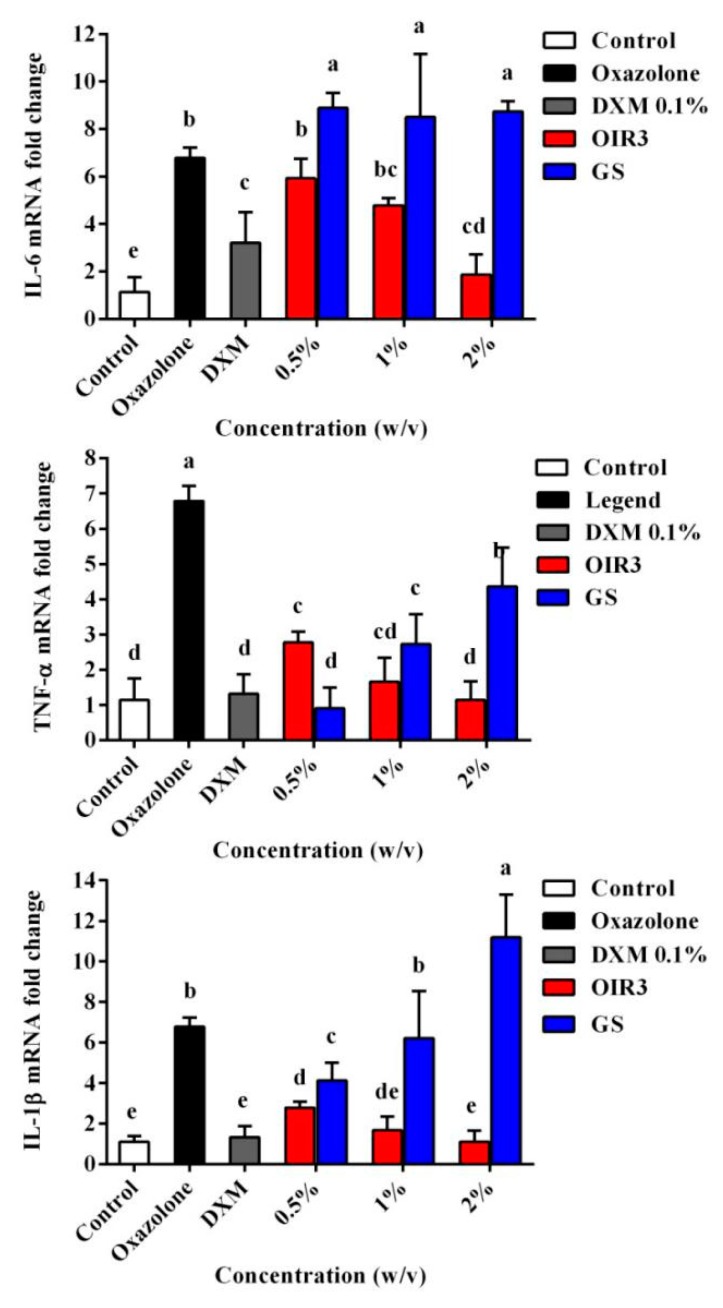
The expression of inflammatory cytokines TNF-α, IL-6 and IL-1β mRNA in mouse ear tissues were determined. Data shown are the mean ± SD of three independent experiments, and a, b, c, d and e represent a significant difference at a level of *p* < 0.05.

We further evaluated the anti-inflammatory activity of OIR3 during oxazolone-induced skin inflammation and compared it to that of GS and DXM. Ear thickness and ear punch weight were significantly increased in the disease model mice, compared to the untreated control mice. OIR3 significantly reduced ear thickness (Figure 12A) and ear punch weight (Figure 12B) in a dosage-dependent fashion, and at the highest concentration of 2% OIR3 produced the lowest ear thickness and ear punch weight, similarly to the group treated with DXM, while GS increased ear thickness and ear punch weight as its concentration increased. Histopathological evaluation of mouse ear tissue demonstrated that prominent epidermal hyperplasia caused by migration of inflammatory cells, including monocytes, granulocytes and macrophages, primarily into the dermis and as well as into the epidermis. According to gross and histological findings, ear edema was remarkably ameliorated in the ears of mice treated with both oxazolone and OIR3 compared to ears of mice treated only with GS (Figure 12C). Furthermore, the expression of mRNA of inflammatory cytokines TNF-α, IL-1β and IL-6 was significantly down-regulated by OIR3 (Figure 13). Sequences used for the amplification primers (5’ to 3’ orientation) were listed in Table 4. These data suggested that OIR3 treatment could effectively attenuate skin inflammation.

## 3. Discussion

Previous studies have shown that the structural changes in AMPs within various environments were related to their biological activities [24,25]. Secondary conformations of these engineered peptides in membrane-mimetic environments were revealed by distinct CD spectroscopic changes. In aqueous solution, most of the engineered peptides assumed an unstructured or random coil-type conformation, while in a membrane-mimetic environment, the active peptides OIR3 and DIR3 adopted a β-hairpin conformation (Figure 1). Conformational rearrangement of the decyclized peptide IR3 vanished in an SDS-containing environment that mimicked the amphiphilic environment within a biological phospholipid bilayer [26], which suggested that the decyclization of a peptide may be strongly associated with its reducing activity (Table 2). In the presence of TFE, D-Pro-containing AMPs DIR1 and DIR2 assumed an obvious β-sheet conformation, which suggested that D-Pro, the optical isomer of L-Pro, may further promote the formation of β-sheets; the propensity for the formation of β-hairpins and β-sheets may be influenced by the presence of alternating linked hydrophobic and hydrophilic amino acids and D-Pro, and this may play an important role in the process of sterilization.

On the one hand, our previous studies found that β-hairpin-like and symmetric-end antimicrobial peptides demonstrated length-dependent antimicrobial activity and membrane-active mechanisms using the quantitative structure activity relationship (QSAR) analysis [27,28]. In this study, the longest of the engineered peptides, OIR3, IR3 and DIR3, each have 14 amino acid residues and exhibit high antimicrobial activity, while the shorter peptides, with the exception of OIR2 and DIR2, inhibit the growth of *E. coli* only slightly and demonstrate no antimicrobial activity against all detected bacteria (Table 2). However, GS, which has 10 amino acid residues, had high antimicrobial activity, which suggested that the length or size of the peptides was not a determinant of the antimicrobial activity [29]. On the other hand, recent studies have showed that cyclization was not essential for the antimicrobial activity of linear and cyclic antimicrobial lipopeptides in the *Paenibacillus* strain [30]; another study found that cyclic peptides do not demonstrate greater permeability than their linear counterparts [29]. However, other studies demonstrated that head-to-tail cyclic peptides had greater antimicrobial activity compared to their linear counterparts [31,32] due to the ability of a head-to-tail cyclic peptide to span the bilayer, facilitating the formation of an anion-selective pore and thereby enabling translocation across the bilayer; in contrast, a linear peptide failed to promote translocation [33]. In this study, the geometric mean of the MICs of the OIR3 peptide was six-fold lower than that of the linear peptide IR3, which indicated that the conformational ordering established upon macrocyclization was associated with an enhanced capacity to inhibit bacterial cell growth. Moreover, the ability of IR3 to adopt a β-hairpin or β-sheet conformation was diminished, likely due the fixed secondary structure produced as a result of the head-to-tail cyclization of the backbone of OIR3 and the presence of Pro that contained rigid β-turns. Previous study found that peptides that including D-form amino acids had higher antimicrobial activity against both Gram-positive and Gram-negative bacteria compared to their counterparts lacking D-form amino acids [34]. DIR3, which contained D-Pro, exhibited higher antimicrobial activity than IR3, which contained only L-Pro, and this may be attributed to the presence of β-hairpins in DIR3 within the simulated membrane environment. An earlier study showed that peptides containing D-type proline displayed a higher affinity for *S. aureus* peptidoglycans and *E. coli* lipopolysaccharides than did those containing L-Pro [34], which suggested that specific interactions between D-form peptides and components of the bacterial cell wall may contribute to elevated antimicrobial activity.

The high cytotoxicity of peptide-based biomaterials towards mammalian cells is a major limitation for the development of AMPs as therapeutics. GS not only has great antimicrobial activity, but it is also highly toxic towards mammalian cells, which is attributed to its amphiphilic structure that causes it to lack selectively in its affinity for cellular membranes [35]. In this study, OIR3, IR3 and DIR3, which each have three IR repeating units, demonstrated no hemolytic activity towards erythrocytes (Table 1) and only weak toxicity towards RAW264.7 cells (Figure 2B), while GS had high toxicity towards mammalian cells, similar to that found previously, suggesting that the simplified, engineered active peptides had a great selectivity for negatively charged bacterial membranes versus zwitterionic mammalian cell membranes. These results demonstrated that the toxicity of AMPs in this system had only a weak relationship with cyclization, linearization and the presence of D-amino acid modification. High hydrophobicity can boost a peptide’s antimicrobial potency, but to some extent it may also induce lysing of both bacteria and human cells, regardless of the degree of membrane damage [36]. GS has two Val (4.1) residues and two Leu residues (9.7), and one Phe (10) that have high hydrophobicity values according to by CCS scale [37] and that are strongly correlated with its greater cytotoxicity. Additionally, two molecules of Ornithine (Orn) with 2 net positive charges caused GS to be poorly amphiphilic. The engineered peptides OIR3, IR3 and DIR3 exhibit greater amphiphilicity, each having 6 net positive charges and 6 hydrophobic residues. Thus, an optimum hydrophobicity-amphiphilicity equilibrium has been suggested for use in the design of rational amphipathicity in peptides [38]. We further determined the cell selectivity of the amphiphiles by calculating their selectivity indexes (SI), which are a measure of the relative safety of the drugs. The cyclic peptide OIR3 had the highest selectivity index, which suggested it had the greatest cell selectivity. The novel finding of this work is that head-to-tail cyclization utilizing the template peptide sequence (IR)_3_P(IR)_3_P (*n* = 1, 2 and 3) produced peptides that had pronounced antimicrobial activity and selectivity enhancing effects.

In addition to the cellular selectivity of antimicrobial peptides, another important challenge for their pharmaceutical development is their stability in the presence of salt ions and serum. Salt ions affect the antimicrobial activity of peptides by interfering with electrostatic interactions and triggering competition in binding between cations and peptides [39]. A previous study demonstrated that the presence of salts would alter electrostatic effects and prohibit cationic peptides from attaching to bacteria, leading to reduced antimicrobial activity [7]. In this study, as shown in Table 3, the presence of Na^+^ and Ca^2+^ slightly compromised the antibacterial actions of peptides against Gram-positive and Gram-negative bacteria, and this may be attributed to weakened electrostatic interactions. The most interesting result, however, was that IR3 had a remarkable ability to resist the effects of salt and demonstrated increased antimicrobial activity against all tested bacteria in high salt conditions. Our previous study showed that the antimicrobial activity of the symmetric-end linear peptide IR2 was increased under high salt conditions, and this may be due to facilitation of the binding of AMPs to bacterial membranes by a relatively low concentration of divalent cations [28]. In this study, the linear peptide IR3 had a more flexible skeletal structure due to it not being restrained by cyclization, which caused it to be more susceptible to salt ions compared to the cyclic peptide OIR3. In addition, OIR3 and DIR3 retained relatively high antimicrobial activity after treatment with serum, suggesting that cyclization and D-Pro modification can increase the stability of peptides in the serum environment.

Overall, the proposed mechanism for the antimicrobial activities of cationic AMPs is the perturbation and destruction of the bacteria membrane via selectively binding cationic peptides to negatively charged components (LPS) exposed within the bacterial membrane via electrostatic interactions; upon reaching a certain concentration threshold, the peptides are then inserted into the bilayer core, where they disrupt lipid packing and create transient pores/ion channels that allow leakage of cellular content across the membrane [40]. LPS, which is an endotoxin, is found at the surface of the outer membrane of Gram-negative bacteria and can regulate the membrane insertion and antibacterial activities of AMPs, depending upon their LPS binding and neutralizing ability [41]. In this study, LPS binding affinities and the SPR assay (Figure 3) demonstrate that OIR3 had a greater endotoxin neutralization potency than IR3, DIR3 and GS. Previous studies showed that, compared to that of their linear counterparts, the constrained backbone of cyclic peptides enhanced their cell permeability [42]. The engineered cyclic peptide OIR3 possessed a higher net positive charge (from six Arg residues) than GS (from two Orn residues), and the bidentate characteristic of the Arg guanidino group is considered to increase binding to the bacterial membrane [43,44]; thus, it is not difficult to infer that OIR3 had greater anti-LPS activity than GS.

The above results are further supported by the fact that OIR3 demonstrates greater outer membrane permeability than IR3, DIR3 and GS at low concentrations (<8 μM), in a concentration-dependent manner. The outer membrane of Gram-negative bacteria is regarded as a major barrier that blocks the translocation of antimicrobial peptides across bacteria membranes [45]. Our results suggested that the large surface area and the reduced conformational flexibility of OIR3 might facilitate its outer membrane permeability (Figure 4). While peptide aggregation increased with increasing peptide concentration (≥8 μM), GS was able to achieve higher outer membrane permeability, which may due to the larger proportion of its hydrophobic amino group that is inserted into the outer membrane. Permeabilization and depolarization of the *E. coli* inner membrane were monitored via β-galactosidase activity using ONPG (Figure 5) and a membrane potential-sensitive fluorescent dye, diSC3-5 (Figure 6). Our data indicates that cyclic peptide OIR3 demonstrated a greater capacity to permeabilize the inner membrane at a concentration >8 μM than IR3, DIR3 and GS. All of the engineered peptides were able to depolarize the cytoplasmic membrane, indicating that the potential dissipation may be correlated with membrane permeability induced by the formation of ionic channels or transmembrane pores. Research had shown that cyclic peptides bind more strongly to negatively charged membranes than linear peptides. The cyclic peptide folds at the membrane interface and adopts a β-sheet structure characterized by two turns, which facilitates its deeper penetration into the bilayer while the linear peptide essentially remains at the surface [46]. Using fluorescence-assisted cell sorting (FACS), the membrane integrity was analyzed and it was further confirmed that OIR3 exhibited an advantage in membrane permeability at 1× MIC (Figure 7). Our SEM (Figure 8) and TEM (Figure 9) studies further showed that OIR3 exerts its bactericidal activity through the destruction of cell membranes via the formation of membrane pores, leakage of the cytosol and eventually lysing of the entire cell [47].

The bacterial flagellum on the surface of Gram-negative bacterial species is primarily a motility organelle that enables motility and chemotaxis, which, in the host, are crucial for the infection of many pathogenic bacteria that must cross the mucosal barrier before accessing the intestinal cells [48]. The bacterial flagellum is driven by flagella motor (a molecular rotary motor) located at the base of the flagella close to the intima of bacterial cells and facing the cytoplasm [49]. In a solid medium containing 0.3% agarose, antimicrobial peptides OIR3, IR3 and DIR3 were able to inhibit bacterial swimming motility, especially at concentrations greater than 4 μM (Figure 10), while GS was unable to inhibit bacterial swimming motility at all tested concentrations. A previous study showed that the motility and the ability of *Salmonella enterica* serovar Typhimurium to invade epithelial cells could be inhibited by an IgA monoclonal antibody that bound to the outermost O antigen of the lipopolysaccharide (LPS) [50], which the major constituent of the outer leaflet of the bacterial outer membrane. In this study, an endotoxin neutralization assay (Figure 3) showed that the cyclic peptide OIR3 had a slightly greater binding affinity for LPS from *E. coli* 25,922 than IR3, DIR3 and the cyclic peptide GS, which suggested that OIR3 may also bind to the O antigen of LPS and act in an antibody-like fashion to inhibit bacterial swimming motility.

The most highly anticipated finding related to the engineered peptides is the immunogenicity produced by the cyclic peptide OIR3 in host cells. Studies have showed that lipopolysaccharides elicit strong inflammatory responses by inducing the overexpression of Toll-like Receptor-4 (TLR4), IκB phosphorylation and degradation, and NF-κB activation, thereby leading to downstream release of early proinflammatory factors such as TNF-α [38]. Antimicrobial peptide AWRK6 significantly inhibited LPS-induced inflammatory responses by blocking the activation of the TLR4/NF-κB pathway [51]. The proinflammatory cytokine TNF-α can cause tissue injury and septic shock, which can lead to pathological conditions such as bacterial and viral infections, autoimmune conditions and inflammatory diseases [52]. Here, the antimicrobial and anti-inflammatory activities of cyclic peptides OIR3 and GS were evaluated by measuring the expression of inflammatory cytokine TNF-α. Upon stimulation of RAW264.7 macrophages with 100 ng/mL LPS in the presence of cyclic peptide OIR3, we found that OIR3 can effectively inhibit TNF-α release in a dosage-dependent method (Figure 11). These results confirmed that the cyclic antimicrobial peptide OIR3 had anti-inflammatory characteristic that inhibit the production of proinflammatory factors.

To further evaluate the anti-inflammatory properties of the engineered peptide OIR3, we also demonstrated the beneficial effects of OIR3 in oxazolone-induced skin inflammation in mice (Figure 12). The cyclic peptide OIR3 potently suppressed oxazolone-induced ear swelling, as demonstrated by its ability to significantly decrease ear thickness and ear punch weight. Histopathological evaluation further confirmed that OIR3 could significantly improve edema of the ear, while GS was not as effective in treating inflammation in the ears of mice. In addition, the cyclic peptide OIR3 improved chronic inflammatory skin disorders, most likely via the inhibition of TNF-α, IL-1β and IL-6 mRNA expression (Figure 13). As previously stated, a cyclic peptide was effective at reducing inflammation in a mouse model of acute colitis, suggesting that the use of cyclic peptides as structural backbones offers a promising approach for the treatment of inflammatory bowel diseases and, potentially, other chronic inflammatory conditions [53]. A recent review reported that host defense peptides can orchestrate host immunomodulatory functions and are able to serve as a bridge between the innate and adaptive immune responses during skin inflammation and infection [54]. Cyclic peptide OIR3 had greater ability to reduce skin inflammation, which may be attributed to its greater abilities to bind and neutralize LPS and inhibit proinflammatory cytokine release, thereby blocking the pathways that induce the expression of inflammatory factors.

## 4. Materials and Methods

### 4.1. Materials

The murine macrophage cell line RAW264.7 was purchased from the cell bank of the Chinese Academy of Sciences, SIBS (Shanghai, China). Mueller Hilton broth (MHB) was obtained from AoBoX (Shanghai, China). Phosphate-buffered saline (PBS) solution was purchased from Solarbio (Beijing, China) and used following dilution to 10 mM (pH 7.4); sodium dodecyl sulfate (SDS) and trifluoroethyl alcohol (TFE) were also purchased from Solarbio (Beijing, China). Human red blood cells (hRBCs) were obtained from healthy blood donors. RPMI 1640 and fetal bovine serum (FBS) were purchased from Invitrogen (Carlsbad, CA, USA). Glucose and lactose (analytical grade) were purchased from Zhiyuan (Tianjin, China). Sodium chloride, potassium chloride, ammonium chloride, zinc chloride, magnesium chloride and ferric chloride were all of analytical grade and purchased from Kermel (Tianjin, China). 3-(4, 5-demethylthiazol-2-yl)-2,5-diphenyltetrazolium bromide (MTT) and dimethyl sulfoxide (DMSO) were purchased from Sigma-Aldrich (Shanghai, China) and Amresco (Wayne, PA, USA), respectively. TritonX-100, propidium iodide (PI), Polymyxin B, o-nitrophenyl-b-D-galactopyranoside (ONPG), N-phenyl-1-napthylamine (NPN), 3.3’-depropylthiadicarbocyanine iodide (DiSC3-5), HEPES, ethanol (analytical grade, 99%), acetone (analytical grade, 99%), lipopolysaccharide (LPS, derived from *E. coli* 055: B5) and BODIPY-TR-cadaverine were all obtained from Sigma (Shanghai, China). Ethanol, acetone, tertiary butanol and glutaraldehyde were all analytical grade (Sigma-Aldrich, Shanghai, China). The Griess reagent system was purchased from Promega (Madison, WI, USA). A mouse TNF-α ELISA kit was purchased from Boster (EK0527, Wuhan, China). Acetate buffer (pH 5.5), M-MLV reverse transcriptase (Takara, Dalian, China), 4-Ethoxymethylene-2-phenyloxazone (oxazolone) and dexamethasone were purchased from Sigma (Shanghai, China).

### 4.2. Bacterial Strains

The test strains, including Gram-negative *E. coli* ATCC25922, *E. coli* ATCC 1005, *S. typhimurium* C7731, *P. aeruginosa* ATCC 27853, *S. Typhimurium* 14028, Gram-positive *S. aureus* ATCC29213, *S. aureus* ATCC 25923, *S. epidermidis* ATCC 12228 and *S. aureus* 43300, were obtained from the School of Veterinary Medicine, Northeast Agricultural University (Harbin, China).

### 4.3. Peptide Design and Sequence Analysis

Head-to-tail cyclic peptides, known as ‘Cyclic Peptide type II’, contain a cyclic backbone with N to C-terminal linkage [55]. In this study, the amphiphilic type II cyclic peptide was constructed according to the template sequence (IR)_n_P(IR)_n_P (*n* = 1, 2 and 3), and the repeat sequences (IR)_n_ were composed of hydrophobic amino acid Ile (I) and hydrophilic amino acid Arg (R). The Ile and Arg were used based on our previous study that found that symmetric-end peptides with IR amino acid repeat sequences had the highest cell selectivity [28]. Arginine contains a guanidinium group, which has a pKa of 12.48, and is therefore always protonated and positively charged during physiological conditions. Arg residues can participate in cation-π interactions, thus potentially enhancing peptide–membrane interactions. Hydrophobic amino acid Ile, which is aliphatic, has a high hydrophobicity according to CCS [37]. Pro, a heterocyclic amino acid, has a side chain with a cyclic structure that locks the φ angle at approximately −65 °C [56] and this often aids in the formation of rigid β-turns due to the lack of a hydrogen-bonding donor group and steric hindrance that prevents hydrogen bonding to adjacent residues. These head-to-tail cyclic peptides adopt a full β-sheet structure where the Ile and Arg residues align to form two antiparallel β-strands, while Ile and Pro form type II′ β-turns. This structure is stabilized by inter-strand hydrogen bonds and thus folds into a fairly rigid amphipathic structure. In addition, to detect the effect of cyclization and modification of the conformation on biological activity, these cyclic peptides were decyclized to obtain linear counterpart peptides IR1, IR2 and IR3. Additionally, we substituted L-Pro with D-Pro in IR1, IR2 and IR3 to create peptides DIR1, DIR2 and DIR3, respectively.

The primary physicochemical parameters and sequences of the engineered peptides were determined using bioinformatics websites, including ProtParam (ExPASy Proteomics Server: http://www.expasy.org/tools/protparam.html) and the antimicrobial peptide database (http://aps.unmc.edu/AP/main.php).

### 4.4. Synthesis and Characterization of Peptides

The engineered peptides were synthesized and purified by the SciLight Biotechnology, LLC company (Beijing, China) using solid-phase methods utilizing N-9-fluorenylmethyloxycarbonyl (Fmoc) chemistry. The molecular weight of the peptides was confirmed using MALDI-TOF MS (Bruker Daltonics Inc., Carlsbad, CA, USA) with α-cyano-4-hydroxycinnamic acid as the matrix. The purity of the peptides was determined to be more than 95% using high-performance liquid chromatography (HPLC). To prevent the degradation of the peptides, each peptide was dissolved in DI water at a concentration of 2.56 mM and stored at −20 °C.

### 4.5. Circular Dichroism (CD) Spectroscopy

Circular dichroism spectroscopy was used to investigate conformational changes induced by the membrane environment [57]. The antimicrobial peptides (AMPs) were dissolved in a 10 mM phosphate buffer solution (PBS; pH 7.4) containing 50% TFE and 30 mM SDS in order to prepare a solution with a certain concentration of the peptide. The circular dichroism spectrometer quartz cell had a light path of 0.1 cm, and the scanning wavelength was 190–250 nm, the resolution was 0.5 nm, the bandwidth (BW) was 1.0 nm and the scanning speed was 50 nm/min. The measurements were carried out at room temperature. The circular dichroism was expressed in terms of the average molar ellipticity, in degrees per centimeter/mole (degree.cm^2^·dmol^−1^).

The acquired CD signal spectra were then converted to the mean residue ellipticity using the following equation:

(1) $$\theta_{M}=\frac{\theta_{obs}\cdot 1000}{c\cdot l\cdot n}$$

where θ_M_ is the mean residue ellipticity (degree·cm^2^·dmol^−1^), θ_obs_ is the observed ellipticity corrected for the buffer at a given wavelength (mdeg), c is the peptide concentration (mM), l is the path length (mm) and n is the number of amino acids.

### 4.6. Antimicrobial Assays

Briefly, the bacteria were incubated overnight at 37 °C with constant shaking at 220 rpm, then were transferred to new MH broth (MHB) medium until they reached the logarithmic phase of growth. The bacteria were then diluted to 10^5^ (CFU)/mL in MHB. The peptides were dissolved and diluted in 0.01% acetic acid and 0.2% bovine serum albumin (BSA) at various concentrations (0.5–128 μM) in each well of sterile 96-well plates and incubated for 20 h at 37 °C. The MIC was determined as the lowest concentration of the peptide that resulted in no bacterial growth, and spectrophotometrically determined by measuring the optical density (OD) at 492 nm at the end of a 20 h incubation. The test was repeated three times.

### 4.7. Stability Assays

The salt ion sensitivity of the peptide was analyzed using the same method used to determine MIC. Determination in *E. coli* ATCC 25,922 and *S. aureus* ATCC 29,213 was conducted at various concentrations of physiological salt ions that simulated in vitro animal body salt concentrations, such as 4.5 mM KCl, 150 mM NaCl, 6 μM NH_4_Cl, 1 mM MgCl_2_, 8 μM ZnCl_2_ and 4 μM FeCl_3_. The stability of the peptide in 50% and 25% human inactivated serum was determined using the same method. The tests were repeated three times.

### 4.8. Hemolysis Assays

The hemolytic activity of the peptides is determined by measuring the amount of hemoglobin released during the lysis of human erythrocytes at different concentrations of AMPs [58]. Briefly, fresh, healthy human red blood cells (hRBCs) were collected and then washed three times with phosphate-buffered saline (PBS) buffer (pH 7.2) and centrifuged at 1000× *g* for 5 min. Each 50 µL sample of hRBCs was incubated with 50 µL of the respective peptide dissolved in PBS in a well of a 96-well plate for 1 h at 37 °C. After incubation, the samples were centrifuged at 1000× *g* for 5 min and the supernatants (50 μL) were transferred to a new 96-well microplate. The release of hemoglobin was measured by monitoring the OD at 570 nm (Tecan, Salzburg, Austria). Negative (hRBCs without AMP treatment) and positive (hRBCs treated with 0.1% TritonX-100) controls were used. The concentration of peptide that caused 5% hemolysis was considered the minimal hemolysis concentration (MHC). The test was repeated three times. The percent hemolysis was calculated using the following formula:

(2) $$\mathrm{Percent}\;\mathrm{hemolysis}=\frac{A-A_{0}}{A_{t}-A_{0}}\times \;100\%$$

A_0_ and A_t_ represent 0% and 100% hemolysis as measured in 10 mM PBS and 0.2% Triton X-100, respectively.

### 4.9. Cytotoxicity Assay

The MTT (3-[4, 5 dimethylthiozol-2-yl]-2,5-diphenyltetrazolium bromide) assay was conducted according to a previously reported MTT colorimetric method [59], and is used as a cell proliferation and cell viability assay. Mouse macrophage RAW264.7 cell lines stored in liquid nitrogen were recovered and cultured in a fully humidified atmosphere with 95% air and 5% CO_2_ at 37 °C. The cells were then diluted, added to 96-well plates at a final concentration of 2 × 10^5^ cells/well, and cultured in RPMI 1640 medium at 37 °C overnight. The next day, peptides were added to the cell cultures at final concentrations of 0.5–128 μM, and untreated cell cultures serving as controls were cultured for 20–24 h in the same conditions. Cell cultures were subsequently incubated with MTT (50 µL, 0.5 mg/mL) for 4 h at 37 °C. The cell cultures were then centrifuged at 1000× *g* for 5 min, and the supernatants were discarded. 150 μL of DMSO was added to dissolve the formazan crystals, and the OD was measured using a microplate reader (Tecan GENios F129004, Salzburg, Austria) at 570 nm. Testing was repeated three times.

### 4.10. Swimming Motility Assays

Since *E. coli* ATCC 25,922 cell walls have flagella, their swimming motility can be measured in LB medium containing 0.3% agar [60]. *E. coli* ATCC 25,922 bacteria were incubated overnight at 37 °C in a shaker until the OD_600_ = 1.0, then inoculated into 0.3% agar medium containing 1 µM, 2 µM, 4 µM or 8 µM of antimicrobial peptide and incubated again overnight at 37 °C. The swimming motility for each was then measured; each experiment was repeated three times.

### 4.11. Binding Affinities to LPS

The BODIPY-TR-cadaverine (BC) displacement assay was previously used to quantify the binding affinities of test compounds to LPS [61]. The fluorescent dye BC is quenched by binding to LPS, causing its fluorescence to disappear. When the antimicrobial peptide is added, if the binding of the peptide to LPS is stronger, BODIPY-TR-cadaverine will be released and the fluorescence restored. Briefly, in a 96-well plate, an equal volume with a different concentration (0.5–64 μM) of peptide was added to a mixture (100 μL) of LPS (25 μg/mL) and BC (2.5 μg/mL) in Tris buffer (pH 7.4), and the background fluorescence was recorded (excitation wavelength 580 nm, emission wavelength 620 nm). The changes in fluorescence were recorded using a Tecan Infinite M200 PRO (Tecan, San Jose, CA, USA); polymyxin B was used as a positive control. Each test was performed independently, in triplicate.

### 4.12. Surface Plasmon Resonance (SPR) Experiments

Surface plasmon resonance (SPR) is a method commonly used to study protein–protein interactions. The main advantage of SPR is that it provides the ability to measure the binding affinities and association/dissociation kinetics of complexes in real time, in a label-free environment, and using relatively small quantities of materials [62]. Real-time binding interactions between peptides and LPS were measured by using the Biacore 3000 instrument (GE Healthcare, Waukesha, Wisconsin, USA). In 10 μg/mL sodium acetate buffer (pH 5.5), the cyclic antimicrobial peptides OIR3, IR3, DIR3 and GS were diluted to an optimum fixed concentration of 25.6 µM, and then covalently immobilized on the CM5 sensor chip; nearly 1100 resonant units (RU) of the peptide were captured. All measurements were performed in 20 mM Tris buffer (pH 7.4) containing 100 mM NaCl. LPS in running buffer (20 mM Tris, 100 mM NaCl, pH 7.4) was allowed to flow over the chip surface at concentrations of 3.125–50 μg/mL at a rate of 30 μL/min. After each injection, the surface was reengineered using a solution containing 20 mM Tris, 50 mM NaOH and 0.05% (*w/v*) SD. Each test was conducted three times independently.

### 4.13. Outer Membrane Permeability Assay

The effect of antimicrobial peptides on the outer leaflet of the cell membrane was examined using the extracellular membrane-sensitive fluorescent dye NPN and the antibiotic-sensitive mutant *E. coli* UB 1005. *E. coli* bacteria were incubated overnight and then transferred to fresh MH broth medium. The bacteria were cultured at 37 °C with shaking at 200 rpm for 3 h until the OD_600_ of the bacterial suspension reached 0.4; then they were centrifuged at 3000 rpm for 3 min and the supernatant was discarded. After adding 5 mM/L HEPES buffer (pH 7.4) that contained 5 mM glucose, the bacteria were collected by centrifugation again. The same buffer was used to suspend bacteria to OD_600_ nm = 0.4; NPN was added to the bacterial suspension at a final concentration of 20 μM. The background fluorescence was measured (excitation λ = 350 nm, emission λ = 420 nm) with an F-4500 fluorescence spectrophotometer (Hitachi, Tokyo, Japan). A 2 mL volume of cell suspension was added to a 1 cm quartz cuvette and mixed with different final peptide concentrations. The fluorescence was recorded in terms of the time needed until there was no further increase in fluorescence after mixing several concentrations (0.5–64 μM) of peptides. Polymyxin B was used as a positive control because of its strong outer membrane permeability. Values were converted to percent NPN uptake using the following equation:

(3) $$\%\mathrm{NPN}\;\mathrm{uptake}=\frac{F_{obs}-F_{0}}{F_{100}-F_{0}}\times \;100\%$$

where F_obs_ is the observed fluorescence at a given peptide concentration, F_0_ is the initial fluorescence of NPN in *E. coli* UB1005 cells and F_100_ is the fluorescence of NPN in UB1005 cells upon the addition of 10 μg/mL polymyxin B, which had strong outer membrane permeability.

### 4.14. Inner Membrane Permeability Assay

The change in intracellular β-galactosidase activity was measured by measuring intracellular β-galactosidase activity [63]. Briefly, *E. coli* UB1005 bacteria were inoculated into MHB containing 2% lactose and cultured overnight. The cells were transferred to new MHB containing 2% lactose and allowed to grow to the logarithmic phase, and then were centrifuged and the bacterial precipitate was collected. The bacteria were resuspended in sterile PBS containing 1.5 mM ONPG to adjust the bacterial concentration to OD_600_ = 0.2; after adding antimicrobial peptide, the absorbance was measured at 420 nm, and the value was read every 2 min. The determination time was 40 min.

### 4.15. Cytoplasmic Membrane Electrical Potential Measurement

We used the cell membrane potential-sensitive fluorescent dye diSC3-5 and the antibiotic-sensitive mutant *E. coli* UB1005 to detect the effect of antimicrobial peptides on cell membrane depolarization [60]. Briefly, mid-logarithmic phase *E. coli* were washed with 5 mM sodium HEPES buffer (pH 7.4) containing 20 mM glucose and resuspended to an OD_600_ = 0.05 in the same buffer. The dye diSC3-5 was added to a final concentration of 0.4 μM until a stable reduction of fluorescence was achieved (approximately 1 h). To equilibrate the cytoplasmic and external K^+^ concentrations, KCl was added to the cell suspension containing diSC_3_-5 to a final concentration of 200 mM, and then incubated for 15 min at room temperature. Of bacterial liquid 2 mL was added to a 24-well plate, and the peptides were added to achieve the desired concentrations (1/4× MIC, 1/2× MIC, 1× MIC, 2× MIC and 4× MIC). The fluorescence was recorded using a Tecan Infinite M200 PRO (Tecan, USA) at an excitation wavelength of 622 nm and an emission wavelength of 670 nm.

### 4.16. Flow Cytometry

Membrane integrity assay. Briefly, *E. coli* ATCC 25,922 were grown to mid logarithmic phase in MHB, washed three times with PBS and diluted to 10^5^ CFU/mL. Different peptides concentrations (1/2× MIC, 1× MIC and 2× MIC) were incubated with the bacterial suspension at a fixed PI concentration of 10 ug/mL for 30 min at 4 °C, and followed by the removal of the unbound dye through washing with PBS. FACS flow cytometer (Becton–Dickinson, Franklin Lake, New Jersey, USA USA) was used to obtain the data with a laser excitation wavelength of 488 nm.

### 4.17. Scanning Electron Microscopy (SEM)

*E. coli* ATCC 25,922 were cultured in MHB to mid-log phase and harvested by centrifugation at 3000× *g* for 5 min. Cells pellets were washed twice with 10 mM PBS and resuspended to an OD_600_ = 0.2. Peptide treatment of the bacterial cells was performed at 37 °C for 1 h at their respective 1× MIC. After incubation, the cells were washed with PBS then centrifuged at 3000× *g* for 5 min three times, and then fixed with 2.5% (*w/v*) glutaraldehyde at 4 °C overnight. The next day, the bacteria were washed twice with PBS and dehydrated using a graded ethanol series (50%, 70%, 90% and 100%) for 15 min each. The samples were then transferred to a mixture (1:1, *v/v*) of ethanol and tertiary butanol, then 100% tertiary butanol, for 20 min each time. After lyophilization and gold coating, the specimens were observed using a scanning electron microscope (Hitachi S-4800, Tokyo, Japan).

### 4.18. Transmission Electron Microscopy (TEM)

Bacterial sample preparation for TEM was conducted using the same method as that for SEM [64]. Bacterial cells were fixed with 2.5% glutaraldehyde at 4 °C overnight, washed twice in PBS, and fixed with 2% osmium tetroxide for 60 min. They were then washed twice with PBS and dehydrated for 5 min each using a graded ethanol series (50%, 70%, 90% and 100%), followed by 8 min each in 100% ethanol and a mixture (1:1) of 100% ethanol and acetone. The sample was then transferred into a 1:1 mixture of absolute epoxy and acetone resin for 35 min, and then into pure epoxy resin for incubation overnight at a constant temperature. The specimens were then sectioned using an ultramicrotome, stained with uranyl acetate and lead citrate, and observed using a HITACHI H-7650 TEM.

### 4.19. Determination of Inflammatory Factors

LPS-activated inflammatory responses are always accompanied by the production of inflammatory cytokines, such as tumor necrosis factor-alpha (TNF-α) [65]. Briefly, 2 × 10^5^ cells/well RAW264.7 cells were plated in 96-well plates and stimulated with LPS (100 ng/mL) in the absence or presence of the peptides (1–32 μM) for 18 h at 37 °C. The levels of TNF-α were determined using an ELISA kit (Boster, Wuhan, China) in accordance with the manufacturer’s instructions. TNF-α was measured by determining the absorbance at 540 nm.

### 4.20. Animal Experiments

ICR mice (male, 4–6 weeks old, and 18–22 g) were obtained from the Experimental Animal Center of Harbin Medical University. All mice were reared in plastic cages with food and water under standard conditions and air filtration (22 ± 2 °C, 12 h light/dark cycles). The study was in accordance with the Local Guide for the Care and Use of Laboratory Animals of Harbin Medical University and used Committee of Northeast Agricultural University (NEAU-(2011)-9).

### 4.21. Allergic Dermatitis Mode

4-Ethoxymethylene-2-phenyl-2-oxazolin-5-one (oxazolone, OXA) was used to induce ear edema in mice [66]. Briefly, all of the experimental mice were randomly divided into eight groups (*n* = 8): 1) the positive control group (0.1% dexamethasone) [67]; 2) the negative control group (without peptide) and 3–8) the treated groups (0.5%, 1% or 2% *w/v* of either OIR3 or GS). The fur was clipped from each animal’s abdomen the day before the experiment was conducted. On the day of the experiment (day 0), 50 ul 3% oxazolone induction solution (prepared in a 5:1 (*v/v*) mixture of acetone: corn oil carrier) was applied to all mice as a single topical application to the shaved abdomen. After 5 days, 50 µL of 1% oxazolone challenge solution (prepared in acetone) was applied as a single topical application to the dorsal area of the right ear for 1 h. Of the peptide solution, vehicle control or positive control (dexamethasone) solution 20 µL was applied topically to the dorsal area of the right ear. The left ear was used as a control. 24 h later, the mice were euthanized by cervical dislocation under ether anesthesia, and two ear punches (6 mm, i.d.) were collected and weighed. The degree of edema and the increase in ear thickness were determined by the increase in the weight and thickness of the right ear punch when compared with that of the left ear. All animal experiments were performed in accordance with the guidelines for the care and use of laboratory animals approved by the Institutional Animal Care and Use Committee of Northeast Agricultural University (NEAU-(2011)-9).

(4) $$\mathrm{Ear}\;\mathrm{weight}\;\mathrm{increase}\;\%=\frac{\left[\mathrm{Right}\;\mathrm{ear}\;\mathrm{weight}\;\left(\mathrm{mg}\right)-\;\mathrm{Left}\;\mathrm{ear}\;\mathrm{weight}\;\left(\mathrm{mg}\right)\right]}{\mathrm{Left}\;\mathrm{ear}\;\mathrm{weight}\;\left(\mathrm{mg}\right)}\times \;100\%$$

(5) $$\mathrm{Increase}\;\mathrm{of}\;\mathrm{the}\;\mathrm{ear}\;\mathrm{thickness}\%\;=\frac{\mathrm{Right}\;\mathrm{ear}\;\mathrm{thickness}\;\left(\mathrm{mm}\right)-\mathrm{Left}\;\mathrm{ear}\;\mathrm{thinkcess}\;\left(\mathrm{mm}\right)}{\mathrm{Left}\;\mathrm{ear}\;\mathrm{thickness}}\times 100\%$$

### 4.22. Hematoxylin and Eosin (HE) Staining

Ear tissues were collected and fixed in 10% formaldehyde for at least 24 h at room temperature. After dehydration in different concentrations of alcohol, tissues were embedded in paraffin and then sliced. H&E staining was performed, and sections were observed under a light microscope (Olympus, Glasgow, UK) [68].

### 4.23. Determination of Inflammatory Factors

Mouse ear tissues were ground in liquid nitrogen and 1 mL of Trizol was added. The total RNA content in each group of ears was extracted using the conventional Trizol method. qRT-PCR was used to measure the mRNA level of inflammatory cytokines TNF-α, IL-6 and IL-1β in mouse ear tissues. One microgram total of RNA was reverse transcribed using M-MLV reverse transcriptase (Takara, Japan). qRT-PCR was performed using a SYBR Green Mix kit (Takara, Japan) with a 7500 Real-Time PCR system according to the manufacturer’s instructions. The mRNA expression levels were normalized to β-actin using the (2^−∆∆*C*t^) method. The gene expression was normalized to the corresponding mGAPDH level. The main primers used for qRT-PCR in this study are shown in Table 4. Each test was repeated at least three times, with three replicates.

### 4.24. Statistical Analysis

Data were analyzed by ANOVA using Graph Pad prism 6.2 software (San Diego, California, USA). Quantitative data are presented as the mean ± standard deviation. *p* < 0.05 was considered to connote statistical significance

## 5. Conclusions

A series of antimicrobial peptides utilizing different types secondary structures, including cyclic, linear, and L-/D-amino acid-modified structures, were designed based on the template (IR)_n_P(IR)_n_P (*n* = 1, 2 and 3) including isoleucine, arginine and proline. In deionized water or a membrane mimetic solution, different secondary structures utilizing cyclization, linearization and L-/D-amino acid modifications exerted different structural transformations, and the most potentially amphiphilic peptide OIR3 exhibited a β-hairpin conformation in a membrane-mimetic environment, while in aqueous solution it formed a random coil. Results in vitro demonstrated that (IR)_3_P(IR)_3_P had the greatest cell selectivity and antimicrobial activity against all measured bacteria, as well as lower erythrocyte cytotoxicity than linear peptide IR3, D-Pro peptide DIR3, or GS. Additionally, cyclic peptide OIR3 exhibited higher stability in various conditions than gramicidin S (GS), which may be attributed to its higher net positive charge and constrained backbone typical of cyclic peptides. The SPR and LPS-binding affinity binding assay indicated that OIR3 exhibited significant endotoxin binding and neutralizing capabilities. In addition, fluorescence spectroscopy, flow cytometry and electron microscopy all showed that OIR3 killed bacterial cells by destroying their cell membranes via cell lysis and the leakage of cytoplasm. LPS-induced inflammation in RAW264.7 cells was used to demonstrate that OIR3 possessed the ability to inhibit the production of pro-inflammatory factor TNF-α. Further evaluation of its anti-inflammatory abilities in vivo revealed that OIR3 exhibited therapeutic effects by inhibiting oxazolone-induced skin inflammation via inhibition of TNF-α, IL-1β and IL-6 mRNA expression. In summary, the engineered head-to-tail cyclic peptide OIR3 had considerable potential as an ideal candidate for use as a clinical therapeutic to treat bacterial infections and skin inflammation.

## Figures and Tables

**Table 1:** Simplified head-to-tail cyclic polypeptide design and key physicochemical parameters.

Peptides	CN ^a^	Sequences	AA	TMW ^b^	MMW ^c^	NC ^d^	RT ^e^ (min)
OIR1	1	Cyclo-(IRPIRP)	6	732.95	732.97	+2	10.32
OIR2	2	Cyclo-(IR)_2_P(IR)_2_P	10	1271.62	1271.66	+4	10.66
OIR3	3	Cyclo-(IR)_3_P(IR)_3_P	14	1810.34	1810.35	+6	12.64
IR1	4	IRPIRP	6	750.94	750.90	+2	10.28
IR2	5	(IR)_2_P(IR)_2_P	10	1289.64	1289.60	+4	10.58
IR3	6	(IR)_3_P(IR)_3_P	14	1828.31	1828.35	+6	12.40
DIR1	7	IR^D^PIR^D^P	6	750.94	750.95	+2	10.44
DIR2	8	(IR)_2_^D^P(IR)_2_^D^P	10	1289.64	1289.60	+4	10.60
DIR3	9	(IR)_3_^D^P(IR)_3_^D^P	14	1828.31	1828.35	+6	12.82

^a^ Compound number. ^b^ Theoretical molecular weight. ^c^ Measured molecular weight (measured by mass spectroscopy). ^d^ Net charge. ^e^ Retention time (RT; min). HPLC retention time was determined using a 4.6 mm × 250 mm Venusil MP C18-5 column (Waters, Milford, MA, USA) at a wavelength of 220 nm with a linear acetonitrile gradient from 15% to 45% in 30 min with buffer A (0.1% trifluoroacetic acid in acetonitrile) and buffer B (0.1% trifluoroacetic acid in water) at a flow rate of 1 mL/min.

**Table 2:** Antibacterial and hemolytic activities of the engineered peptides and antibiotics.

Strains	MICs ^a^ (µM)												
	OIR1	OIR2	OIR3	IR1	IR2	IR3	DIR1	DIR2	DIR3	GS	CH ^b^	CS ^c^	PS ^d^
Gram-negative													
*E. coli* 25922	>128	64	2	>128	>128	16	>128	128	4	4	16	0.5	128
*E. coli* UB 1005	>128	64	2	>128	>128	8	>128	128	4	4	4	0.5	64
*P. aeruginosa* 27853	>128	>128	8	>128	>128	8	>128	>128	8	4	>128	0.5	128
*S. typhimurium* 7731	>128	>128	4	>128	>128	16	>128	>128	4	8	8	0.5	0.5
*S. typhimurium 14028*	>128	>128	4	>128	>128	16	>128	>128	16	16	16	4	16
Gram-positive													
*S. aureus* 29213	>128	>128	4	>128	>128	64	>128	>128	8	2	8	64	64
*S. aureus* 25923	>128	>128	4	>128	>128	64	>128	>128	64	2	32	64	32
*MRSA*43300 ^e^	>128	>128	4	>128	>128	64	>128	>128	64	2	32	64	32
*S. epidermidis* 12228	>128	>128	4	>128	>128	>128	>128	>128	128	2	32	128	0.5
nGM (–) ^f1^	256	147.0	3.5	256	256	12.1	256	194.0	6.1	6.1	18.4	0.8	24.3
pGM (+) ^f2^	256	256	4.0	256	256	90.5	256	256	45.3	2	22.6	76.1	13.5
tGM ^f3^	256	188.1	3.7	256	256	22.6	256	219.5	14.8	3.7	20.2	5.9	18.7
MHC_5_^g^	>128	>128	>128	>128	>128	>128	>128	>128	>128	8	>128	>128	>128
nSI (–) ^h1^	1.0	1.7	73.1	1.0	1.0	21.2	1.0	1.3	42.0	1.3	13.9	320.0	10.5
pSI (+) ^h2^	1.0	1.0	64.0	1.0	1.0	2.8	1.0	1.0	5.7	4.0	11.3	3.4	19.0
tSI ^h3^ [20,21]	1.0	1.4	69.2	1.0	1.0	11.3	1.0	1.2	17.3	2.2	12.7	43.4	13.7

^a^ Minimum inhibitory concentrations (MICs) were determined as the lowest concentration of peptide that prevented visible turbidity. ^b^ Chloramphenicol. ^c^ Colistin Sulfate. ^d^ Penicillin Sodium. ^e^ Methicillin-resistant *Staphylococcus aureus*. ^f1^ nGM (–) was the geometric mean of the peptide MICs against gram-negative strains tested. ^f2^ pGM (+) was the geometric mean of the peptide MICs against gram-positive strains tested. ^f3^ tGM was the geometric mean of the peptide MICs against all the 9 bacteria tested. ^g^ MHC_5_ was the minimum hemolytic concentration that that induced ≥5% hemolysis of human red blood cell. When no detectable hemolytic activity was observed at 128 μM, a value of 256 μM was used to calculate the selectivity index. ^h1^ nSI (−) was the selectivity index that was calculated as MHC_5_/GM (−). ^h2^ pSI (+) was the selectivity index that was calculated as MHC_5_/GM(+). ^h3^ tSI was the total selectivity index that was calculated as MHC_5_/TGM.

**Table 3:** MIC^a^ values of the engineered peptides in the presence of physiological salts and serum.

Peptides	Control ^b^	NaCl ^b^	KCl ^b^	NH_4_Cl ^b^	MgCl_2_^b^	CaCl_2_^b^	ZnCl_2_^b^	FeCl_3_^b^	Serum ^c^	
Gram-negative strain *E. coli* ATCC 25922									25%	50%
OIR3	2	4	2	2	2	4	2	2	4	8
IR3	16	32	16	16	16	32	16	16	32	64
DIR3	4	16	16	8	8	16	8	8	8	16
GS	4	16	4	4	8	16	4	4	32	64
Gram-positive strain *S. aureus* ATCC 29213										
OIR3	4	8	4	4	4	8	4	4	8	16
IR3	64	32	16	16	16	32	16	16	32	64
DIR3	8	16	8	8	8	16	8	8	8	16
GS	2	2	4	2	2	2	2	2	32	>64

^a^ Minimum inhibitory concentrations (MIC) were determined as the lowest concentration of the peptides that inhibited bacteria growth. ^b^ The final concentrations of NaCl, KCl, NH_4_Cl, MgCl_2_, CaCl_2_, ZnCl_2_, and FeCl_3_ were 150 mM, 4.5 mM, 6 μM, 1 mM, 2 mM, 8 μM, and 4 μM, respectively, and the control MIC values were determined in the absence of these physiological salts (salt ion concentration in normal human blood). ^c^ Human blood serum was inactivated by heat treatment for 15 min at 56 °C. MICs in the presence of 25% and 50% human blood serum were determined.

**Table 4:** Sequence of the amplification primers in the 5’ to 3’ orientation.

Target Gene	Sequence (5′–3′)
TNF-α	Forward: CCTATGTCTCAGCCTCTTCTCAT
	Reverse: CACTTGGTGGTTTGCTACGA
IL-6	Forward: GGAGAGGAGACTTCACAGAGGA
	Reverse: ATTTCCACGATTTCCCAGAGA
IL-1β	Forward: TGAAATGCCACCTTTTGACAG
	Reverse: CCACAGCCACAATGAGTGATAC
mGAPDH	Forward: TGTTCCTACCCCCAATGTGT
	Reverse: TGTGAGGGAGATGCTCAGTG

## Supplementary Materials

Supplementary materials can be found at https://www.mdpi.com/1422-0067/20/23/5904/s1.

## Abbreviations

AMPs	antimicrobial peptides
CD	circular dichroism
DMSO	dimethyl sulfoxide
EDTA	ethylenediaminetetraacetic acid
FACS	fluorescence-assisted cell sorting
FBS	fetal bovine serum
hRBCs	human red blood cells
LPS	lipopolysaccharides
MHB	Mueller-Hinton broth
MIC	minimum inhibitory concentration
MTT	3-(4,5-dimethylthiozol-2-yl)-2,5-diphenyltetrazolium bromide
NF-κB	nuclear factor-κB
NO	nitric oxide
NPN	N-phenyl-1-naphthylamine
OD	optical density
PI	propidium iodide
qRT-PCR	quantitative real-time PCR
SDS	sodium dodecyl sulfate
SEM	scanning electronic microscopy
TFE	trifluoroethyl alcohol
TLR4	Toll-like receptor-4
DXM	dexamethasone
