# Supplementary material for: Simplified Head-to-Tail Cyclic Polypeptides as Biomaterial-Associated Antimicrobials with Endotoxin Neutralizing and Anti-Inflammatory Capabilities
Source: Int J Mol Sci. 2019 Nov 25;20(23):5904. doi: 10.3390/ijms20235904 (PMC6928678; doi:10.3390/ijms20235904)
Supplement: Supplementary file 1 [file ijms-20-05904-s001.pdf]

International Journal of *Molecular Sciences*

MDPI

Supplementary Materials:

Simplified Head-to-Tail Cyclic Polypeptides as Biomaterial-Associated Antimicrobials with Endotoxin Neutralizing and Anti-Inflammatory Capabilities

Na Dong [2], Chensi Wang [2], Xinran Li [2], Yuming Guo [1,*] and Xiaoli Li [3]

[1] State Key Laboratory of Animal Nutrition, College of Animal Science and Technology, China Agricultural University, Beijing 100193, China
[2] The Laboratory of Molecular Nutrition and Immunity, Institute of Animal Nutrition, Northeast Agricultural University, Harbin, 150030, China; ndong@neau.edu.cn (N.D.); s1650059155@gmail.com (C.W.); zl15713822260@gmail.com (X.L.)
[3] Heilongjiang Key Laboratory of Molecular Design and Preparation of Flame Retarded Materials, College of Science, Northeast Forestry University, Harbin, 150040, China; lixiaoli0903@nefu.edu.cn
* Correspondence: guoyum@cau.edu.cn; Tel.: (86-010)-6273-3900

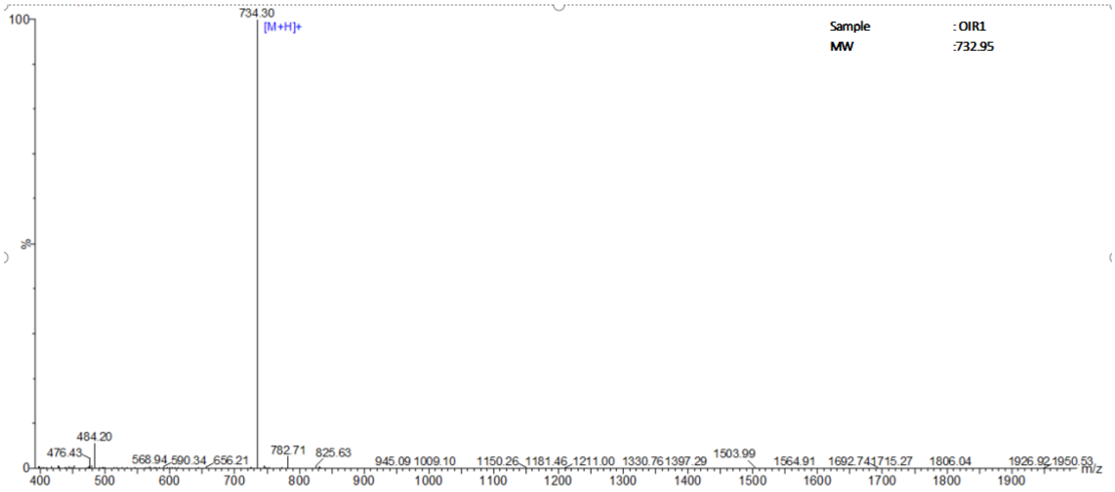
Sample : OIR1
MW :732.95
734.30
[M+H]+
484.20
476.43
782.71
825.63
1503.99
%
m/z

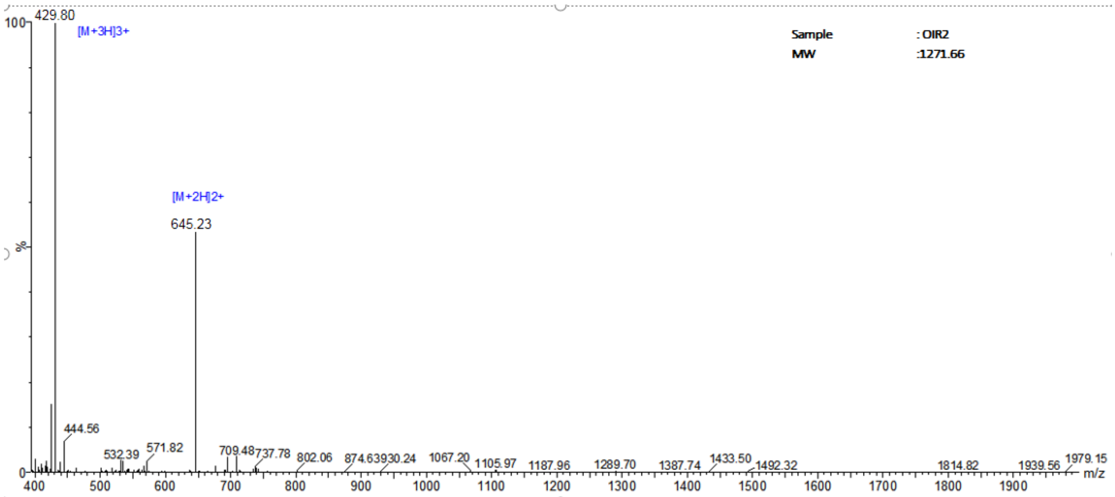
Sample : OIR2
MW :1271.66
429.80
[M+3H]3+
[M+2H]2+
645.23
444.56
532.39
571.82
%
m/z

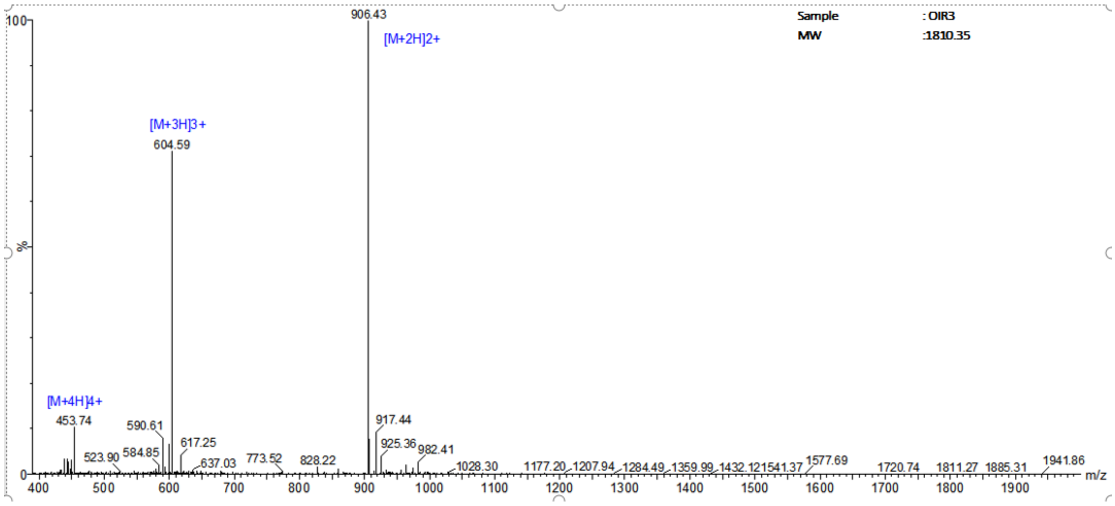
Sample : OIR3
MW :1810.35
906.43
[M+2H]2+
[M+3H]3+
604.59
[M+4H]4+
453.74
590.61
617.25
917.44
925.36
982.41
%
m/z

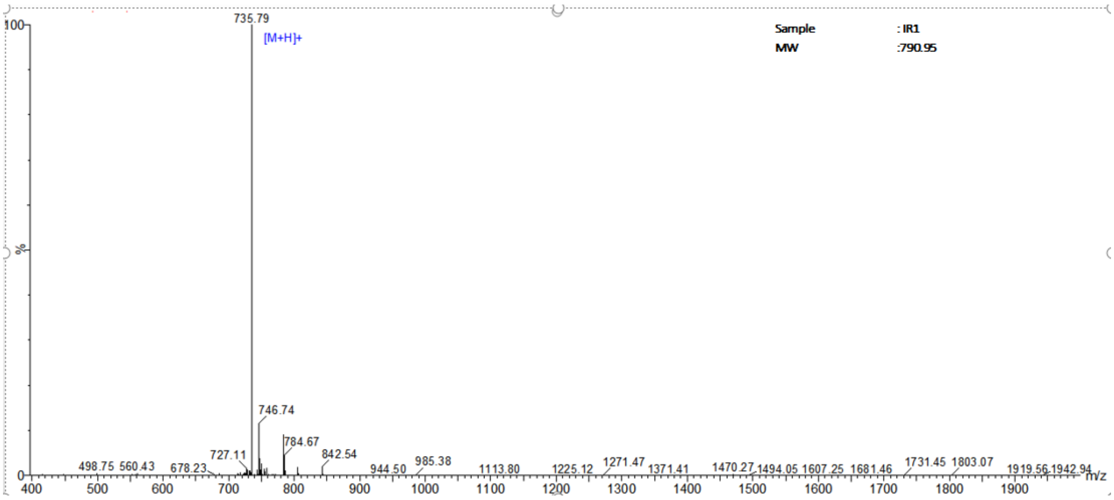

Sample : IR1
MW :790.95
735.79
[M+H]+
746.74
784.67
842.54
727.11
498.75 560.43 678.23
944.50 985.38
1113.80
1225.12 1271.47 1371.41
1470.27 1494.05 1607.25 1681.46
1731.45 1803.07
1919.56 1942.94
%
m/z

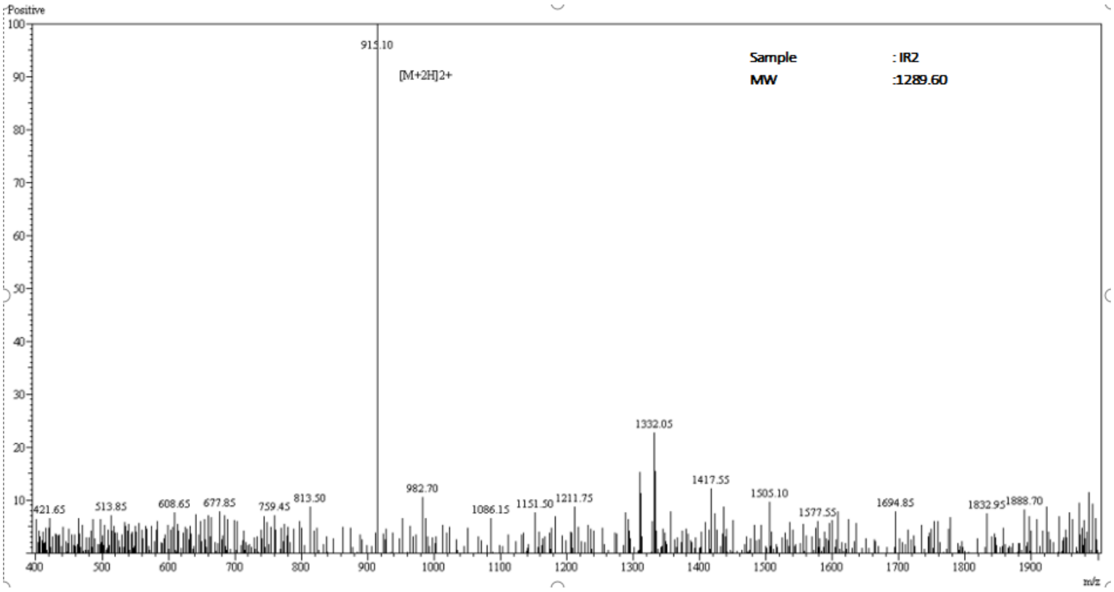

Positive
Sample : IR2
MW :1289.60
[M+2H]2+
1332.05
1417.55
1505.10
982.70
421.65 513.85 608.65 677.85 759.45 813.50
1086.15 1151.50 1211.75
1577.55 1694.85 1832.95 1888.70
m/z

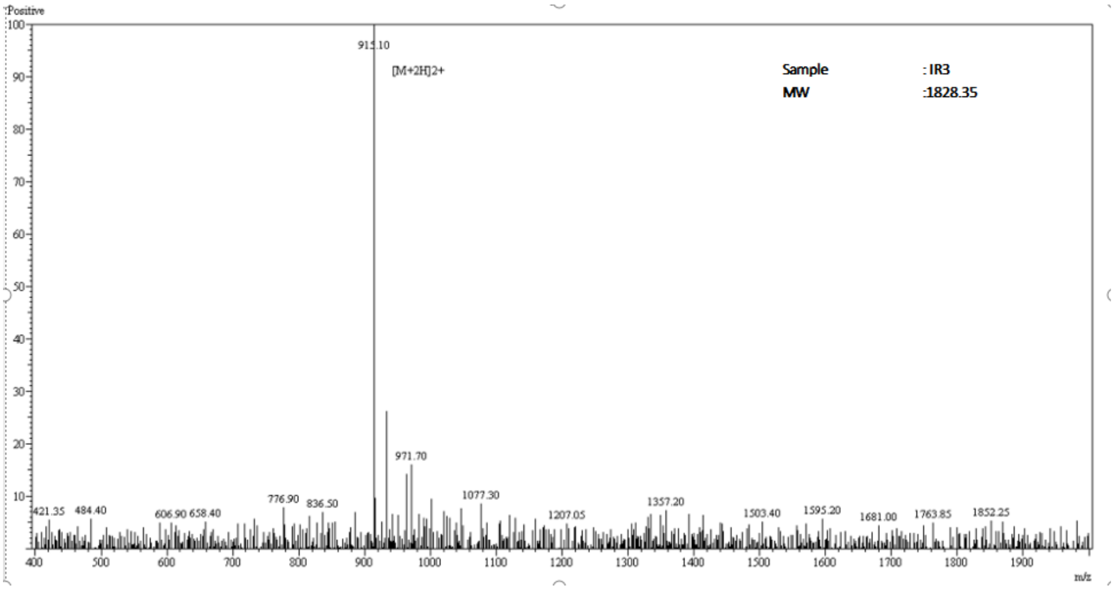

Positive
Sample : IR3
MW :1828.35
[M+2H]2+
971.70
1077.30
421.35 484.40 606.90 658.40 776.90 836.50
1207.05 1357.20 1503.40 1595.20 1681.00 1763.85 1852.25
m/z

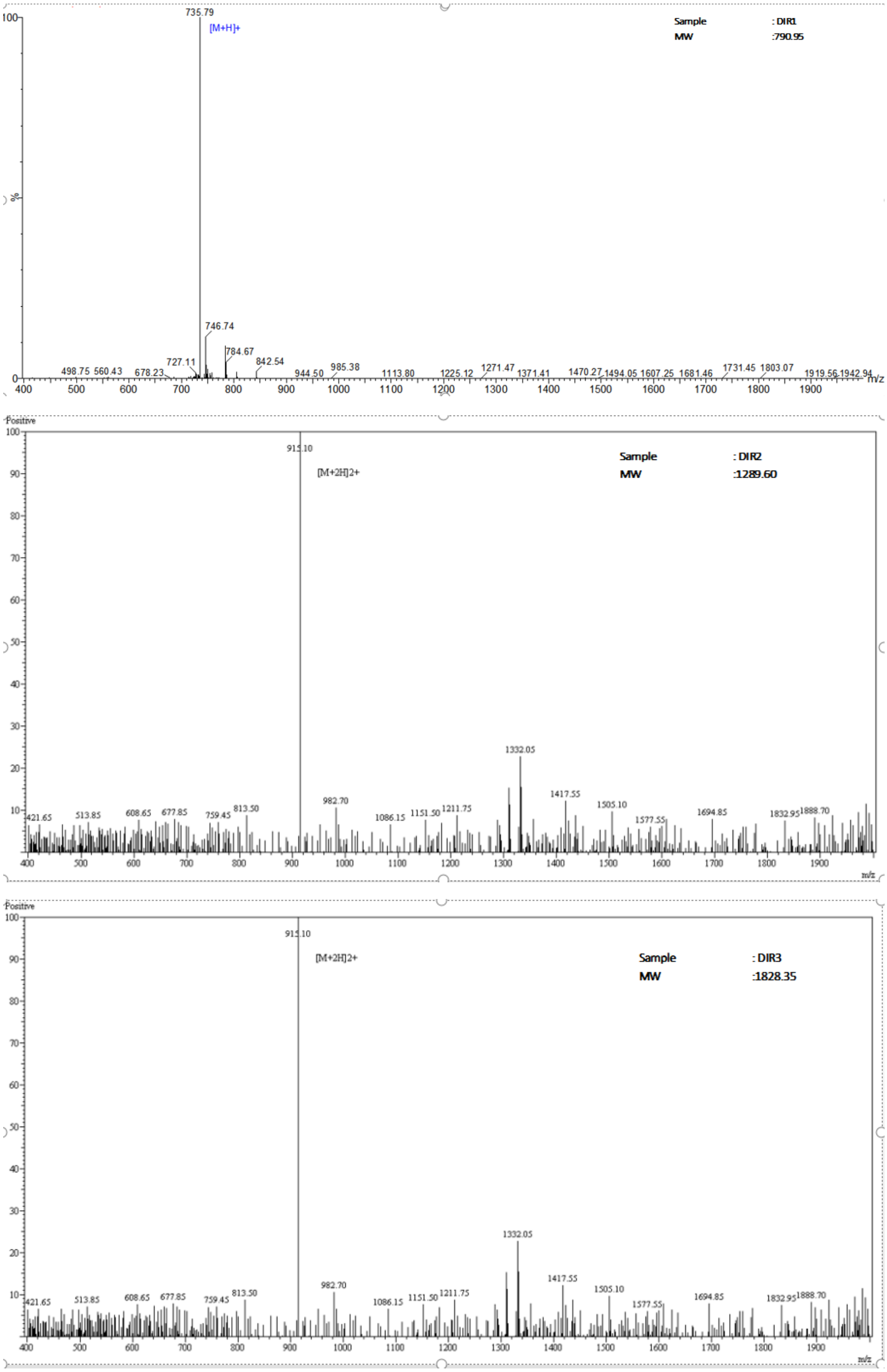

Figure S1. MALDI-TOF MS of the engineered peptides.
